# A novel 10-gene ferroptosis-related prognostic signature in acute myeloid leukemia

**DOI:** 10.3389/fonc.2022.1023040

**Published:** 2022-10-20

**Authors:** Kai Zhu, Zhichao Lang, Yating Zhan, Qiqi Tao, Zhijie Yu, Lili Chen, Congcong Fan, Yan Jin, Kang Yu, Bihan Zhu, Yuxiang Gao, Chengchi Wang, Songfu Jiang, Yifen Shi

**Affiliations:** ^1^ Key Laboratory of Diagnosis and Treatment of Severe Hepato-Pancreatic Diseases of Zhejiang Province, The First Affiliated Hospital of Wenzhou Medical University, Wenzhou, China; ^2^ Department of Hematology, The First Affiliated Hospital of Wenzhou Medical University, Wenzhou, China; ^3^ Zhejiang Provincial Clinical Research Center For Hematological disorders, Wenzhou, China

**Keywords:** ferroptosis, TCGA database, AML, Prognosis signature, Immune infiltration, machine learning

## Abstract

Acute myeloid leukemia (AML) is one of the most common hematopoietic malignancies and exhibits a high rate of relapse and unfavorable outcomes. Ferroptosis, a relatively recently described type of cell death, has been reported to be involved in cancer development. However, the prognostic value of ferroptosis-related genes (FRGs) in AML remains unclear. In this study, we found 54 differentially expressed ferroptosis-related genes (DEFRGs) between AML and normal marrow tissues. 18 of 54 DEFRGs were correlated with overall survival (OS) (P<0.05). Using the least absolute shrinkage and selection operator (LASSO) Cox regression analysis, we selected 10 DEFRGs that were associated with OS to build a prognostic signature. Data from AML patients from the International Cancer Genome Consortium (ICGC) cohort as well as the First Affiliated Hospital of Wenzhou Medical University (FAHWMU) cohort were used for validation. Notably, the prognostic survival analyses of this signature passed with a significant margin, and the riskscore was identified as an independent prognostic marker using Cox regression analyses. Then we used a machine learning method (SHAP) to judge the importance of each feature in this 10-gene signature. Riskscore was shown to have the highest correlation with this 10-gene signature compared with each gene in this signature. Further studies showed that AML was significantly associated with immune cell infiltration. In addition, drug-sensitive analysis showed that 8 drugs may be beneficial for treatment of AML. Finally, the expressions of 10 genes in this signature were verified by real-time quantitative polymerase chain reaction. In conclusion, our study establishes a novel 10-gene prognostic risk signature based on ferroptosis-related genes for AML patients and FRGs may be novel therapeutic targets for AML.

## Introduction

Acute myeloid leukemia (AML) is characterized by a heterogeneity of molecular abnormalities and the accumulation of immature myeloid progenitors in the bone marrow and peripheral blood and represents the most common type of acute leukemia in adults (1, 2). Despite novel treatment options over the last years, the 5-year survival rate of AML patient remains unsatisfactory (3). 40%~70% of AML patients relapse and become treatment-refractory, ultimately leading to treatment failure and even death. Therefore, there is an urgent need to develop novel prognostic biomarkers to monitor the prognosis of AML patients.

Ferroptosis is an iron-dependent form of regulated cell death driven by a lethal increase of lipid peroxidation (4, 5). Ferroptosis has been shown to play a key role in the suppression of tumorigenesis by removing the cells deficient in key nutrients in the environment or damaged by infection or ambient stress (6). Targeting ferroptosis is considered as a promising way for cancer patients, especially for malignancies that are resistant to traditional treatments (7, 8). Several signatures with ferroptosis-related genes have been established to predict the prognosis of patients with cancer (9). However, the role of FRGs in the prognosis of AML remains unclear.

In this study, we constructed a prognostic signature of 10 ferroptosis-related differentially expressed genes (FRDEGs) based on the transcriptomic and clinical data of AML patients from The Cancer Genome Atlas (TCGA). Then, this FRDEGs prognostic signature was validated by International Cancer Genome Consortium (ICGC) and the First Affiliated Hospital of Wenzhou Medical University (FAHWMU) cohorts. Using functional enrichment analysis and correlation analysis, we further explored the potential molecular mechanisms in our signature. Finally, we performed a drug sensitivity analysis to explore potential gene targets.

## Materials and methods

### Data collection

The RNA sequencing (RNA-seq) and clinical data of two AML cohorts were downloaded from public database, including 130 tumor samples (bone marrow) of AML patients from TCGA (https://portal.gdc.cancer.gov) and 92 tumor samples (bone marrow) of AML patients from ICGC (https://dcc.icgc.org/projects/LIRI-JP). Besides, RNA-seq data of 70 normal marrow samples were obtained from Genotype-Tissue Expression Project (GTEx) (https://www.genome.gov/). All the expression data from the three databases were normalized using the perl, respectively. The current research follows the TCGA and ICGC data access policies and publication guidelines. A total of 60 FRGs utilized in this study were obtained from the previous literature (**Supplementary Table 1**) (7).

In addition, we collected 57 tumor samples (bone marrow) of AML patients from the FAHWMU as validation data.

### Construction of a prognostic 10-gene signature

The “limma” R package was used to identify the DEGs between tumor samples from TCGA and normal samples from GTEx with a false discovery rate (FDR)< 0.05 (**Supplementary Table 2**). Moreover, with the help of the “survival” R package, we assessed the prognostic values of 60 FRGs and calculated their FDRs using the Benjamin–Hochberg (BH) method. Protein-Protein Interaction Networks (PPI) and correlation networks of the intersecting 18 genes were generated using the STRING database (STRING: functional protein association networks (string-db.org)). Least absolute shrinkage and selection operator (LASSO) Cox regression was performed using the “glmnet” R package. The independent variable in the regression was the normalized expression matrix of candidate prognostic differentially expressed genes, and the response variables were overall survival (OS) and status of patients in the TCGA cohort. The optimum penalty parameter (λ) for the model was determined by 10-fold cross-validation following the minimum criteria (i.e. the value of λ corresponding to the lowest partial likelihood deviance). The riskscore of the patients was calculated according to the normalized expression level of each gene and its corresponding regression coefficients. The formula was established as follows:


$${\text{score= e}}^{\text{sum (expression level of each gene × corresponding coefficient)}}$$

Patients were stratified into the high- or low-risk groups based on the median value of their risk score. Patients in the ICGC were also stratified into the high- and low-risk groups based on the values derived from this formula.

### Validation of a prognostic 10-gene signature

Based on the expression levels of genes in the signature, we carried out Principal Component Analysis (PCA) using the “prcomp” package. Besides, t-distributed Stochastic Neighbor Embedding (t-SNE) was performed to explore the clustering of different groups using the “Rtsne” R package. Univariate and multivariate Cox regression analyses were used to identify independent prognostic factors. Receiver Operating Characteristic (ROC) curve analysis was used to predict OS with the R package “pROC”. All statistical analyses were carried out using the R software, with P< 0.05 being considered statistically significant.

### Machine learning method analysis for 10-gene signature

SHapley Additive explanation (SHAP) was used to explore the importance of 10 genes and riskscore for the 10-gene signature. SHAP (10) is a game theory method that interprets machine-learning model and understands the decision-making process through quantifying the contribution that each feature brings to the prediction made by the model.

### Functional enrichment and correlation analysis

The “clusterProfiler” R package was utilized to conduct Gene Ontology (GO) and Kyoto Encyclopedia of Genes and Genomes (KEGG) analyses based on DEGs (**Supplementary Table 3**, |log2FC| ≥ 1, FDR< 0.05) between the high- and low- groups from TCGA cohort. P values were adjusted using the BH method. Moreover, we estimated the infiltration score of 16 immune cell types and the activity of 13 immune-related pathways using single-sample gene set enrichment analysis (ssGSEA) in the “gsva” R package. Besides, based on the Expression data (ESTIMATE) algorithm, we estimated the proportion of infiltrating immune cells and stromal cells to get immune, stromal and ESTIMATE score for each AML patient. Using CIBERSOFT algorithm, the relative content score of 22 TICs in every AML patient was calculated. CIBERSOFT is a gene-based deconvolution algorithm that infers 22 human tumor immune infiltrating cell types and quantifies (11). The Cancer Stem Cell (CSC) correlation analysis and tumor microenvironment correlation analysis were conducted using the “limma” and “estimate” R packages.

### Drug sensitivity analysis

The CellMiner website (https://discover.nci.nih.gov/cellminer/) was used to analyze the NCI-60 database (12, 13). The target gene expression status and z-score for cell sensitivity data were retrieved from the website and analyzed using Pearson correlation analysis to evaluate the relationship between target gene expression and drug sensitivity.

### Quantitative real-time PCR analysis

The bone marrow samples of AML patients (n=20) as well as healthy donors (n=20) were collected from the FAHWMU. Total RNA was isolated from AML patients as well as healthy donors using the Tiangen RNA extraction reagent kit. Each sample was reversely transcribed into complementary DNA (cDNA) using a reverse-transcription (RT) reagent kit (Takara Biotechnology Co., Ltd., Dalian, China). Then, Real-time PCR was performed using SYBR Premix ExTaq (Takara). GAPDH was used as endogenous controls for mRNAs. The primer sequences for 10 genes were shown in **Supplementary Table 4**.

### Statistical analysis

R software (version 4.0.3) and GraphPad prism 9 were used to complete all statistical work and plot drawing. The Spearman correlation method was employed to calculate the correlation between two variables. Survival plots were created using the Kaplan–Meier method. Two sets of data for qRT-PCR were analyzed using Student’s t-test. To examine the relationship between OS and riskscore as well as clinical feature, univariate or multivariate Cox regression analysis was performed. The hazard ratio (HR) and 95% confidence interval (CI) were calculated to identify genes associated with OS. P< 0.05 was considered statistically significant.

## Results

### Flow chart and clinical data

**Figure 1:**
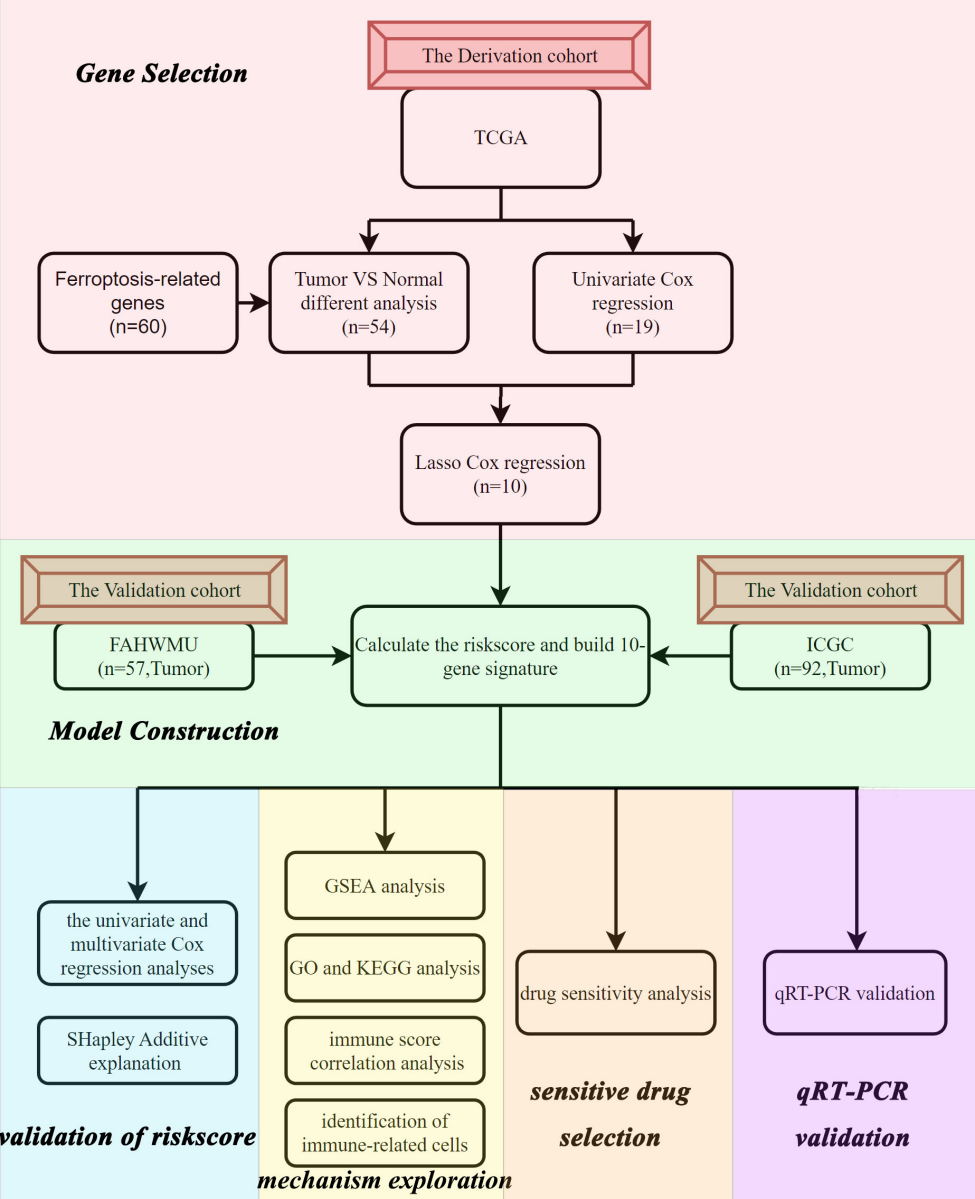
Flow chart of the data collection and analysis.

The flow chart of this study was shown in **Figure 1**. Data from a total of 130 AML tumor samples from the TCGA cohort and 92 ICGC tumor samples derived from AML patients were used. Detailed clinical characteristics of patients were summarized in **Table 1**.

**Table 1:** Clinical characteristics of AML patients used in this study.

	TCGA cohort	ICGC cohort
**No.of patients**	130	92
**Age (median,range)**	56 (21-88)	62 (18-88)
**Gender (%)**		
**male**	70	49
**Female**	60	43
**Stage (%)**		
**M0**	12 (9.2%)	NA
**M1**	30 (23%)	NA
**M2**	32 (24.6)	NA
**M3**	14 (10.8%)	NA
**M4**	27 (20.8)	NA
**M5**	12 (9.2%)	NA
**M6**	2	NA
**M7**	1	NA
**Survival status (%)**		
**Alive**	52 (40%)	0
**Dead**	78 (60%)	92 (100%)
**Survival time (median)**	364 days	303 days

NA, Not Applicable.

### Identification of prognostic 18 FRDEGs in the TCGA cohort

**Figure 2:**
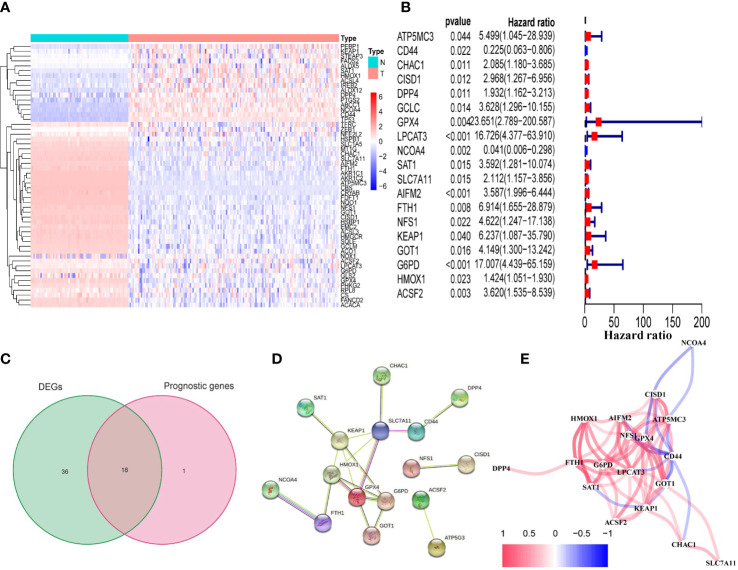
Identification of prognostic FRDEGs in the TCGA cohort. **(A)** Heat map of the 54 DEGs (T: tumor sample; N: normal sample). **(B)** Forest plots showed the results of the univariate cox regression analysis between gene expression and OS (p < 0.05). **(C)** Venn diagram identified FRDEGs. **(D)** The PPI network revealed the hub genes. **(E)** The correlation network among the 18 DEFRGs genes. Different colors represent the correlation coefficients.

We found that the majority of FRGs were differentially expressed between TCGA tumor samples and GTEx normal samples (54/60, 90%). Eighteen of these FRDEGs (**Figures 2B** **, C**) were associated with OS in univariate Cox regression analysis (p<0.05, **Figure 2A**). Using PPI network construction, we identified the hub genes including SLC7A11, G6PD, GPX4, HMOX1, and FTH1 (**Figure 2D**). The correlation among 18 FRDEGs was shown in **Figure 2E**.

### 10 FRDEGs were selected and 10-gene signature was constructed in the TCGA cohort

10 of 18 prognostic FRDEGs, which were determined by LASSO Cox regression* *analysis, were selected for the next analysis (**Supplementary Figures 1A, B** **)**. A riskscore was calculated using mRNA expression levels and relevant coefficients of 10 genes with the following formula:


Riskscore= (-0.548∗CD44)+(0.371∗CHAC1)+(0.629∗CISD1)+(0.399∗DPP4)+(-0.849∗NCOA4)+(0.299∗SAT1)+(0.485∗SLC7A11)+(0.280∗AIFM2)+(1.391∗G6PD)+(0.955∗ACSF2)

### Survival analyses of this 10-gene signature in TCGA, ICGC and FAHWMU cohorts

**Figure 3:**
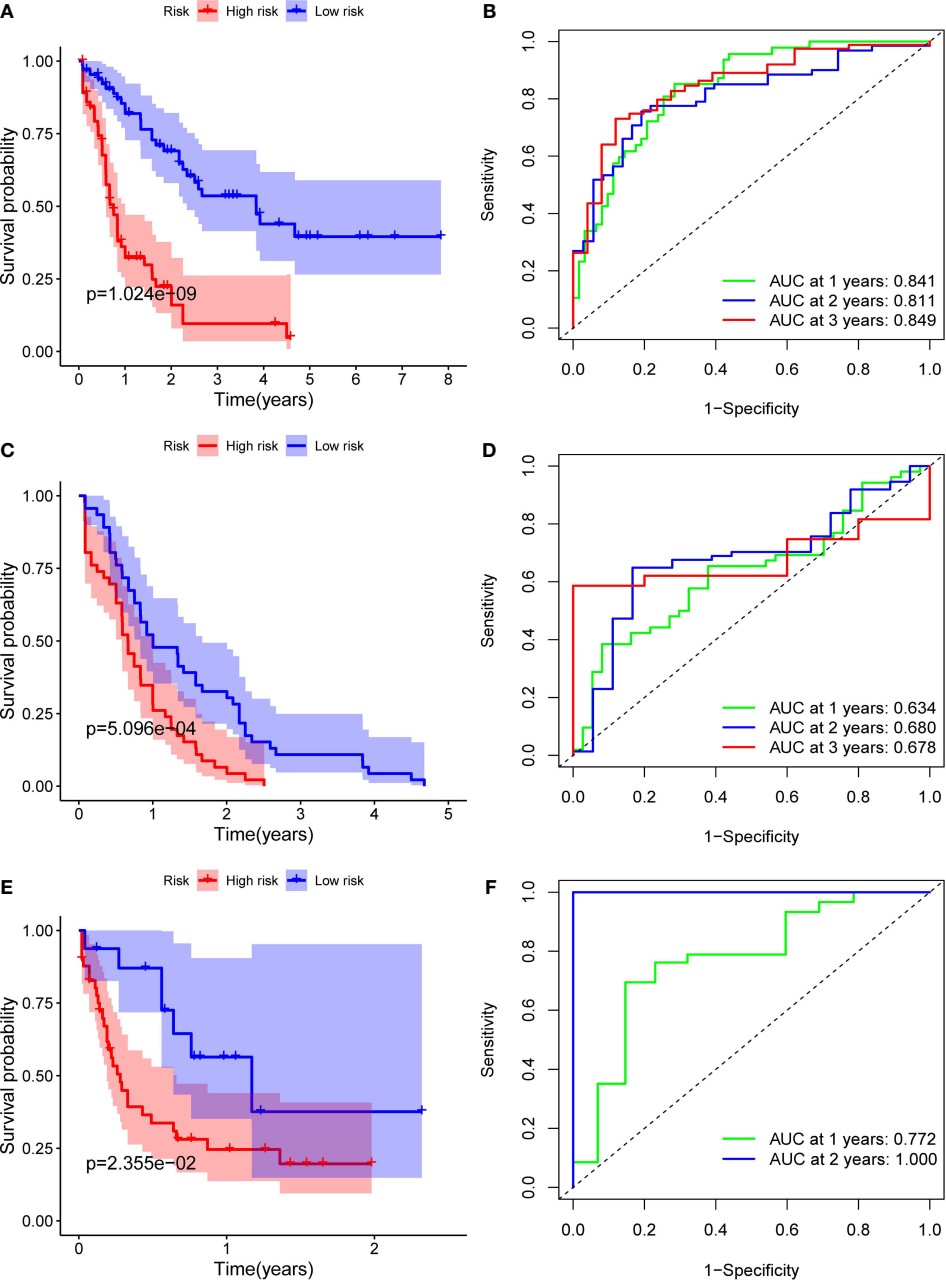
Identification of ten optimal FRGs. **(A)** Kaplan-Meier curves for the OS of patients in the high-risk and low-risk groups in the TCGA cohort. **(B)** AUC of time-dependent ROC curves verified the prognostic performance of the riskscore in the TCGA cohort **(C)** Kaplan-Meier curves in the ICGC cohort. **(D)** AUC of time-dependent ROC curves in the ICGC cohort. **(E)** Kaplan-Meier curves in the FAHWMU cohort. **(F)** AUC of time-dependent ROC curves in the FAHWMU cohort.

**Figure 4:**
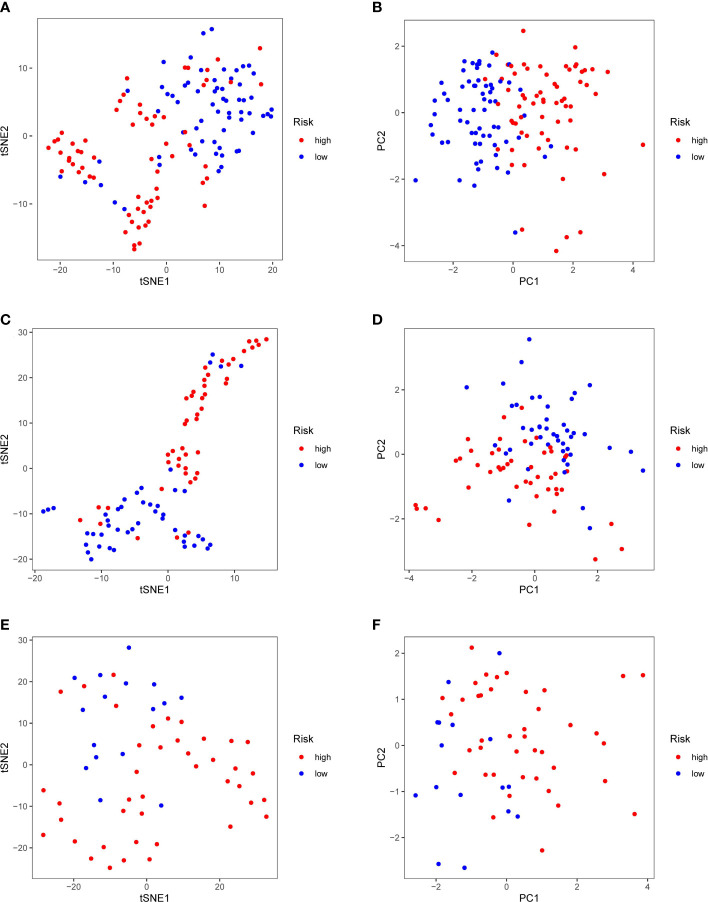
PCA and t-SNE analysis. tSNE **(A)** and PCA **(B)** dimension reduction analysis of the high- and low-risk groups based on the riskscore in TCGA cohort. tSNE **(C)** and PCA **(D)** dimension reduction analysis of the high- and low-risk groups based on the riskscore in ICGA cohort. tSNE **(E)** and PCA **(F)** dimension reduction analysis of the high- and low-risk groups based on the riskscore in FAHWMU cohort. (high: high-risk group; low: low-risk group).

Patients in the TCGA, ICGC or FAHWMU cohort were then divided into the high- or low-risk groups according to the median cut-off value. The results of Kaplan-Meier curve indicated that patients in the low-risk group exhibited a significantly better OS than those in the high-risk group in TCGA (**Figure 3A**, P<0.001), ICGC (**Figure 3C**, p<0.001) and FAHWMU cohorts (**Figure 3E**, P<0.05). The predictive performance of this riskscore for OS was evaluated by time-dependent ROC curves. In the TCGA cohort, the area under the curve (AUC) reached 0.841 for 1^st^ year, 0.811 for 2^nd^ year, and 0.849 for 3^rd^ year (**Figure 3B**). In the ICGC cohort, the AUC was 0.634 for 1^st^ year, 0.680 for 2^nd^ year, and 0.678 for 3^rd^ year (**Figure 3D**). The AUC of 10-gene signature in the FAHWMU cohort was 0.772 for 1^st^ year and 1.000 for 2^nd^ year, respectively (**Figure 3F**). The t-SNE and PCA plots, mapped based on the risk score of each patient, were shown in **Figures 4A–F**. The red point means patient in the high-risk group, while blue point means patient in the low-risk group (**Figures 4A-D**). It was found that the red points clustered in one part, while the blue points clustered in another part. Results of this outcome suggest that our 10-gene signature may contribute to better prognosis prediction of AML patients.

### Identification of independent prognostic value

**Figure 5:**
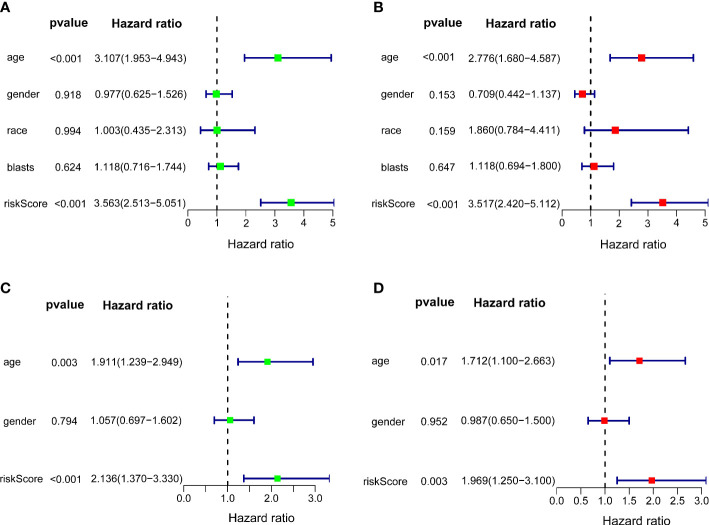
Independent prognostic value of the 10-gene signature. Forest plots of univariate cox regression analyses **(A)** and multivariate cox regression analyses **(B)** in TCGA cohort. Forest plots of univariate cox regression analyses **(C)** and multivariate cox regression analyses **(D)** in the ICGC cohort.

Univariate and multivariate Cox regression analyses were carried out among the available variables to determine whether the riskscore was an independent prognostic predictor for OS. In TCGA cohort, the riskscore was significantly associated with OS in both the univariate Cox regression analyses (HR = 3.563, 95% CI = 2.513-5.051, P< 0.001) (**Figure 5A**) and multivariate Cox regression analyses (HR = 3.517, 95% CI = 2.420-5.112, P< 0.001) (**Figure 5B**). Similar results including both univariate Cox regression analyses (HR = 2.136, 95% CI = 1.370-3.330, P< 0.001) (**Figure 5C**) and multivariate Cox regression analyses (HR = 1.969, 95% CI = 1.250-3.100, P = 0.003) (**Figure 5D**) were also found in the ICGC cohort. Except for the riskscore, age is another character that was identified as the independent prognostic factors (P<0.05)

### Machine learning method determines the importance of each feature

**Figure 6:**
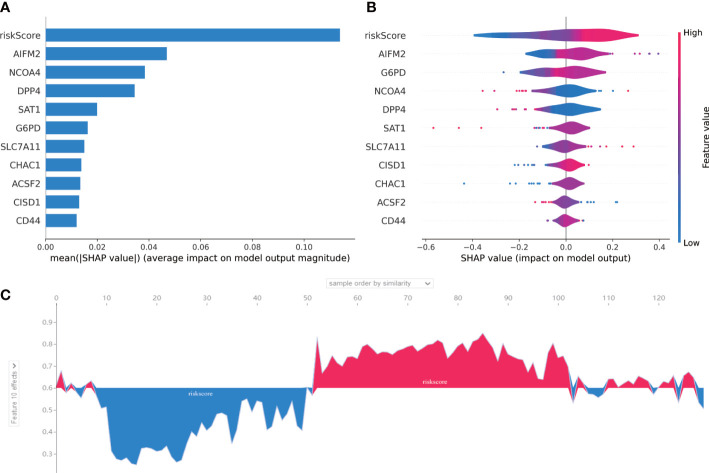
SHAP for 10-gene signature. **(A)** A score calculated by SHAP was used for each input feature. **(B)** The contribution of each input feature in the overall model. When the SHAP value is positive, if the Future value is mainly red, this feature is a positive correlation. **(C)** The performance of the riskscore in AML prognosis was assessed by SHAP. The abscissa is to sort each patient according to the riskscore from low to high, and the ordinate is the SHAP value for each patient. Blue represents patient survival and red represents patient death.

In order to judge the importance of each feature in our 10-gene signature, we used SHAP method. As shown in **Figure 6A**, riskscore had the highest correlation with this 10-gene signature compared with each gene in this signature. In addition, AIFM2 was found to have high contribution. Riskscore was positively correlated with our 10-gene signature and AIFM2 was shown to be negatively associated this signature (**Figure 6B**). As indicated by **Figure 6C**, our riskscore was shown to have a good predictive effect on the patient’s survival status.

### GO and KEGG analysis of DEGs in the high- and low-risk groups

**Figure 7:**
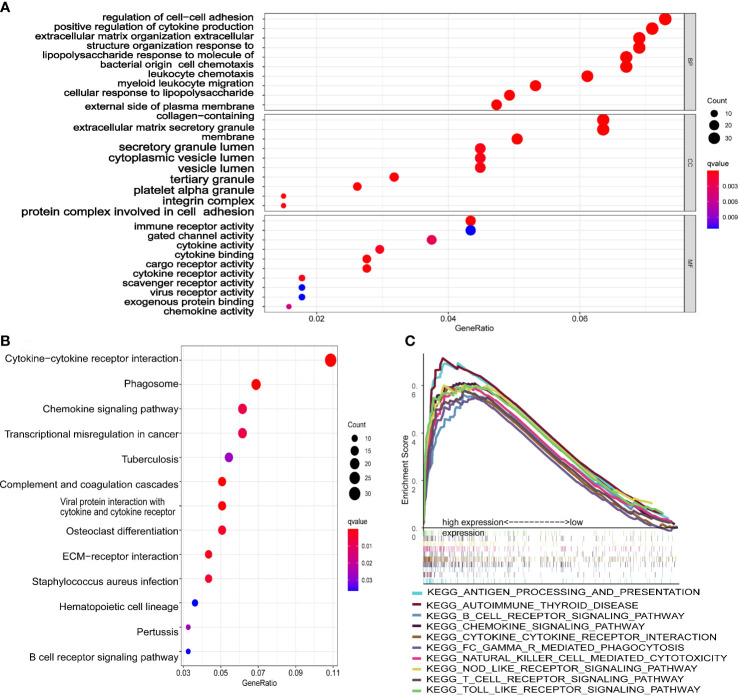
Functional analysis of the 10-gene signature. **(A)** The GSEA plot of top 10 enriched pathways. **(B)** GO enrichment analysis (*p* < 0.05, *q* < 0.05; BP, biological process; CC, cellular component; MF, molecular function). **(C)** KEGG enrichment analysis (*p* < 0.05, *q* < 0.05).

GO analysis (**Figure 7A**) showed that DEGs were significantly involved in the biological processes of extracellular structure (matrix, external side of plasma membrane collagen, cell−cell adhesion), the cellular components that occur in cytoplasmic lumen and some immune-related function (leukocyte chemotaxis, leukocyte chemotaxis, immune receptor, cytokine). The pathways of the KEGG database (**Figure 7B**) indicated that DEGs were significantly involved in the immune and stromal related-pathway (phagosome, chemokine, viral protein interaction with cytokine and cytokine receptor, ECM−receptor interaction). The results of GO and KEGG revealed that DEGs may play a key role in the prognosis and immune-related response in AML patients.

### Immune infiltration was associated with the riskscore of 10-gene signature

**Figure 8:**
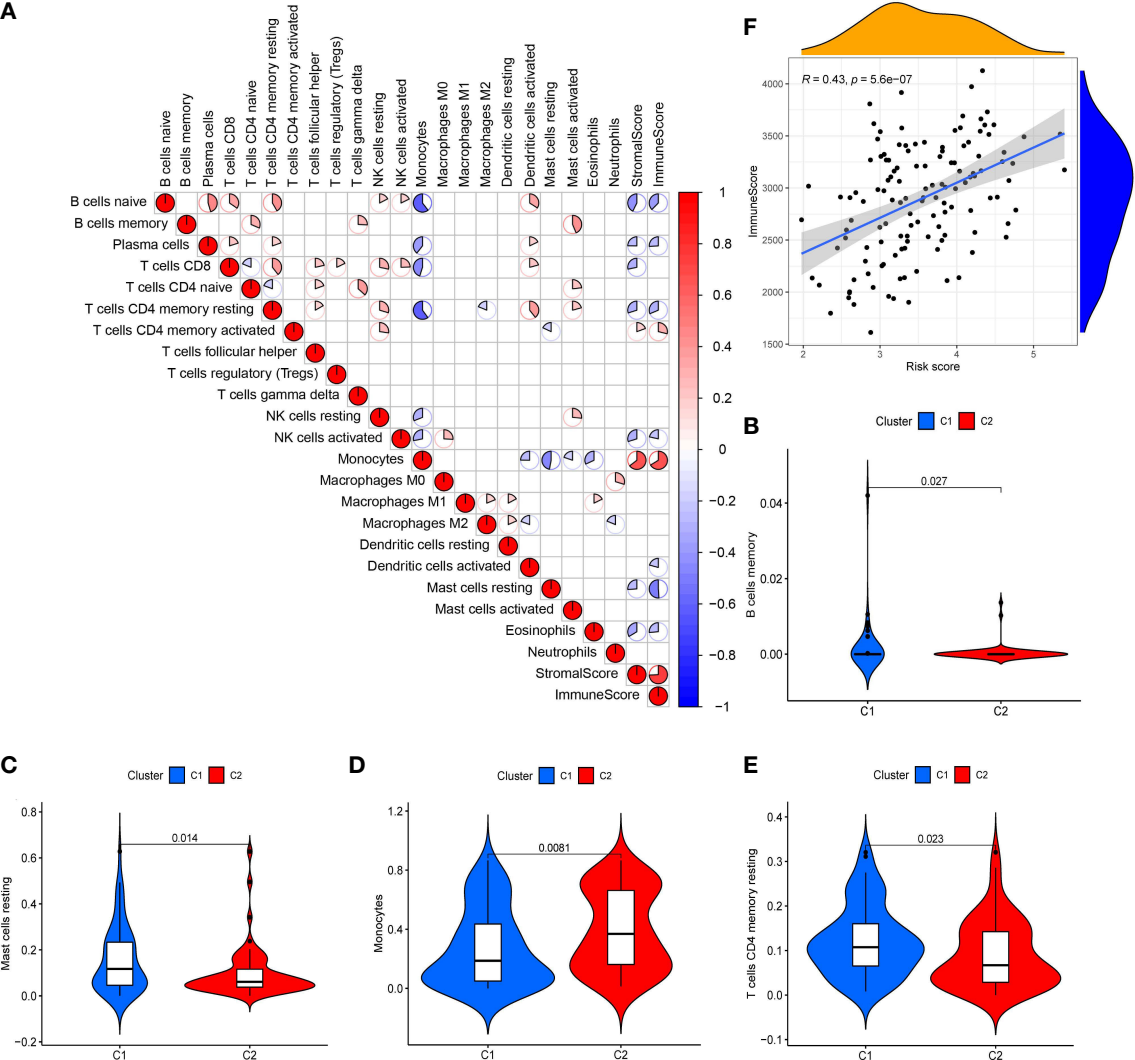
Analysis of tumor microenvironment in 10-gene signature **(A)** Correlation plots of 18 immune-related cells (derived from CIBERSOFT algorithms), stromal scores and immune score (derived from ESTIMATE algorithms) in AML (red: positive correlation; blue: negative correlation; p < 0.05). **(B-E)** Violin diagrams of 4 immune-related cells (B cells memory, Monocytes, T cells CD4 memory and Mast cells). **(F)** Scatter plot of the correlation between immune score and the riskscore in the 10-gene signature.

The scatter plots were used to explore the association between the tumor microenvironment and the riskscore. As shown in **Figure 8F**, the immune score was positively correlated with riskscore (p<0.0001; R=0.43). GSEA analysis indicated that the top 10 pathways of DEGs between the high- and low-risk groups were involved in the biological processes of immune response (antigen processing and presentation, B cell receptor signaling pathway, chemokine signaling pathway, cytokine-cytokine receptor interaction, Fc gamma r mediated phagocytosis, natural killer cell mediated cytotoxicity, NOD-like receptor signaling pathway, T cell receptor signaling pathway, Toll like receptor signaling pathway) (**Figure 7C**). Therefore, we further explored the correlations between 18 immune-related cells and the riskscore *via* CIBERSOFT algorithm. 8 types of immune-related cells (naive B cells, Plasma cells, T cells CD4 memory, NK cells, Monocytes, Dendritic cells, Mast cells and Eosinophils) were correlated with immune score (**Figure 8A**, p<0.05). There were positive correlations between immune score and T cells CD4 memory as well as Monocytes and Mast cells. Subsequently, 4 types of cells (B cells memory, Monocytes, T cells CD4 memory, Mast cells, p<0.05) were selected for further correlation analysis between the high- and low-risk groups (**Figures 8B–E**). The expressions of Monocytes in the high-risk group were higher than that in the low-risk group. The expressions of T cells CD4 memory and Mast cells were lower than that in the low-risk group. However, no significant difference in the expressions of B cell memory between the high- and low-risk groups. Our data indicate that the immune infiltration is significantly related with the riskscore.

### AML patients may be sensitive to 8 drugs

**Figure 9:**
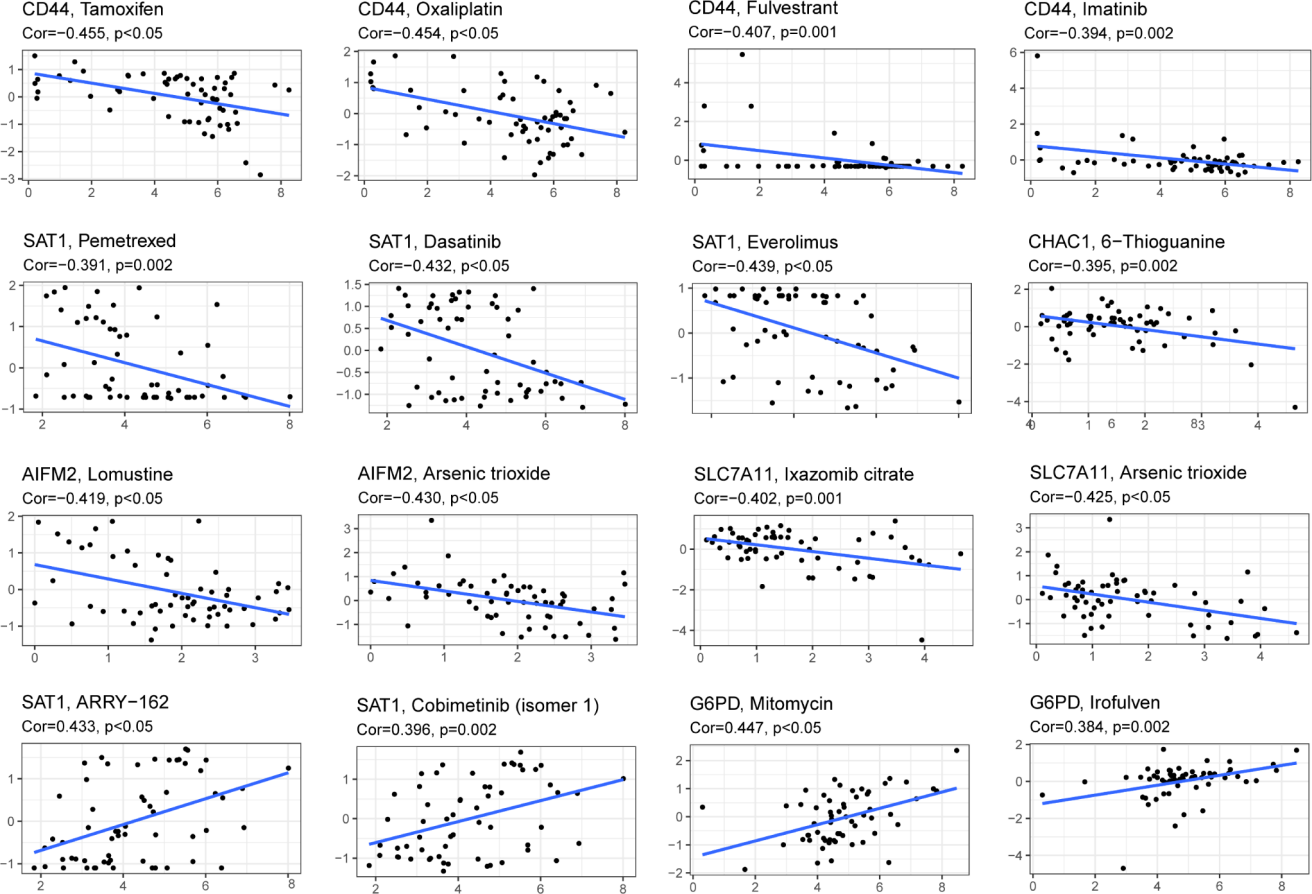
Drug sensitivity analysis of the 10-gene signature. (Cor > 0 means that patients with high expression of this gene may be sensitive to this drug; Cor< 0 means that patients with high expression of this gene may be resistant to this drug. P < 0.05).

Drug sensitivity analysis was used to identify potential drugs that AML patients may be sensitive. Drug sensitivity analysis was analyzed between the top 16 drugs and 10 genes. Only the results of drug sensitivity analysis with P<0.05 were shown in **Figure 9**. There were positive correlations between 4 drugs (ARRY-162, Cobimetinib, Mitomycin and lrofulven) and SAT1 as well as G6PD in AML patients from the TCGA cohort. In addition, there were negative correlations between 4 drugs (Tamoxifen, Oxaliplatin, Fulvestrant and lmatinib) and CD44. Combined with these, AML patients with dysregulation of SAT1, G6PD or CD44 may be sensitive to 8 drugs (ARRY-162, Cobimetinib, Mitomycin, lrofulven, Tamoxifen, Oxaliplatin, Fulvestrant and lmatinib).

### Validation of expressions of 10 genes of this 10-gene signature in AML

QRT-PCR was performed to validate the mRNA expression levels of 10 genes in our signature in the FAHWMU cohort. As shown in **Supplementary Figure 2**, up-regulated CD44, DPP4, SAT1 and NCOA4 were found in AML patients, while CHAC1, CISD1, SLC7A11, AIFM2, G6PD, and ACSF2 were down-regulated in AML patients.

## Discussion

AML patients have been reported to benefit from advances in targeted molecular and immunotherapy (14, 15), however, the 5-year survival rate of AML patients remains unsatisfactory due to high relapse rates. Stratification of patients into the high- and low-risk groups based on reliable molecular signatures may aid in selecting appropriate treatment strategies in line with precision medicine. Emerging studies have indicated the vital roles of FRGs in tumorigenesis (16–18). However, the relationship between AML prognosis and FRGs remains unclear. In this study, we established a novel ferroptosis-related prognostic gene signature for AML patients. We assessed the relationships between 60 FRGs and OS, and subsequently identified 18 FRDEGs. Using LASSO Cox regression, we selected 10 of 18 FRDEGs for construction of a prognostic gene signature. We also compared enrichment score of infiltration of immune cells and immune pathways between the high- and low-risk groups, investigated functional mechanisms *via* GSEA, and assessed potentially suitable drugs. This novel 10-gene signature may contribute to the improvement in the prediction of AML prognosis and patient stratification for therapeutic strategies.

The FRGs (CD44, CHAC1, CISD1, DPP4, NCOA4, SAT1, SLC7A11, AIFM2, G6PD, and ACSF2) were included in our 10-gene signature. CD44, a cell-surface glycoprotein, has been reported to be involved in cell-cell interaction, cell adhesion, and migration (19). Previously, it has been demonstrated that CD44 expression is closely related with the occurrence of tumors, including AML (20–22). Stevens et al. found that CHAC1 contributes to the inhibition of AML *via* atovaquone (23). Inhibition of CISD1 results in iron accumulation and oxidative injury in mitochondria, thus contributing to erastin-induced ferroptosis in hepatocellular carcinoma cells (24). In B-cell acute lymphoblastic leukemia, CHAC1 can overcome drug resistance and exert anti-leukemic activity (25). Loss of TP53 prevents nuclear accumulation of DPP4 and thus facilitates plasma-membrane-associated DPP4-dependent lipid peroxidation, resulting in ferroptosis (26). CARS1 has been included in a novel prognostic signature by Chen et al., which effectively predicts the prognosis of Clear Cell Renal Cell Carcinoma (27). Activation of SAT1 induces lipid peroxidation and sensitizes cells to undergo ferroptosis upon reactive oxygen species (ROS)-induced stress (28). Inactivation of SLC7A11 has a synergistic effect with APR-246 for the promotion of cell death (29). G6PD has previously been proposed as a biomarker for AML (30). A recent study has revealed a potential relationship between AIFM2 and EBF3, which acts as a tumor suppressor gene in AML (31). ACSF2 participates in the regulation of the lipid metabolism *via* peroxisome proliferator-activated receptor alpha. Recently, Wang et al. constructed a FRG signature for breast cancer patients, which included ACSF2 (32). All the 10 genes are associated with ferroptosis process and the prognosis of tumors, especially AML.

Recently, immune infiltration has been reported to be involved in the progression of AML. For example, Luca et al. found that the bone marrow immune environment of AML patients is profoundly altered (33). A previous study demonstrated that a higher level of B and T cell activation was found in AML samples than non-tumor samples (34). NK cells can trigger the anti-leukemia responses (35) and ferroptosis has been shown to exert anti-tumor immune effects by triggering dendritic cell maturation (36). Therefore, we explored the association between immune cell infiltration and the riskscore in this study. Our data revealed that higher Monocytes levels were found in the high-risk group. In addition, Mika T et al. found that high expression of Monocytes is related to the failure of the first induction therapy in AML (37), which indicated that AML patients in the high-risk group with higher expression of Monocytes may be relate to the worse OS. Besides, it has been found that differentiated monocyte-like AML cells express diverse immunomodulatory genes and suppress T cell activity *in vitro* (38). In this study, the high-risk group with lower level of T cells CD4 memory was associated with the higher counting of differentiated monocyte-like AML cells, which may be responsible for the bad prognosis of the high-risk group. Besides, in our tumor microenvironment correlation analysis, the riskscore was positively associated with the immune score. Our findings revealed an association between the 10-gene signature and immune cell infiltration.

In the past few decades, targeted cancer therapies have developed rapidly. However, treatment of AML remains unsatisfactory (39). In this study, we performed drug sensitivity analysis to find AML drugs that may have clinical benefits. We selected 8 drugs (ARRY-162, Cobimetinib, Mitomycin, lrofulven, Tamoxifen, Oxaliplatin, Fulvestrant and lmatinib) that AML patients with dysregulation of SAT1, G6PD or CD44 may be sensitive to. Among them, ARRY-162, Cobimetinib, Mitomycin, lrofulven, Tamoxifen and Oxaliplatin have already been reported to be applied in AML patients for clinical trials or cells (40–45). Lmatinib has been authorized to treat chronic myeloid leukemia (CML) since 2001 (46). No reported have been found in the treatment of Fulvestrant in AML. Our drug sensitivity analysis provides novel promising drugs for AML patients, and more studies are still needed for further validation in the future.

Recently, several risk signatures of AML have been established based on FRGs (47–49). However, our study still has many advantages. Firstly, we firstly reported a novel prognostic risk signature of 10 FRGs for AML based on the data from TCGA and ICGC cohort. Secondly, we validated this 10-gene signature in a local cohort (FAHWMU cohort). Thirdly, this signature revealed an association between FRGs and immune cell infiltration in AML. In addition, we used a machine learning method to validate our 10-gene signature. Finally, we found 8 potential drugs for AML clinical treatment in the future. However, there are many limitations in our research. A single hallmark (ferroptosis) was used to construct a prognostic model, which may lead to the loss of many key prognostic genes of AML. In addition, the detailed roles of FRGs in AML including *in vivo* and *in vitro* should be further explored in the future.

## Conclusion

Collectively, our study establishes a novel 10-FRG prognostic risk signature for AML patients. In addition, FRGs may represent novel therapeutic targets in AML.

## Data availability statement

The original contributions presented in the study are included in the article/**Supplementary Material**. Further inquiries can be directed to the corresponding authors.

## Ethics statement

The studies involving human participants were reviewed and approved by the Human Research Ethics Committee in The First Affiliated Hospital of Wenzhou Medical University. Written informed consent was obtained from the individual(s) for the publication of any potentially identifiable images or data included in this article.

## Author contributions

KZ, ZL and YZ conceived the project and wrote the manuscript. LC, CF, QT, ZY, BZ, YG, CW, and KY participated in data analysis. SJ and YS participated in discussion and language editing. All authors contributed to the article and approved the submitted version.

## Funding

The project was supported by the Natural Science Foundation of Zhejiang Province (No. LQ19H080002), and the Public Welfare Science and Technology Project of Wenzhou (No. Y20190119). Public Welfare Science and Technology Project of Wenzhou (No. Y20220028).

## Acknowledgments

We thank the TCGA, ICGC, and GTEx databases for providing valuable datasets. This manuscript was submitted as a pre-print in the link https://www.researchsquare.com/article/rs-980809/v2 (50).

## Conflict of interest

The authors declare that the research was conducted in the absence of any commercial or financial relationships that could be construed as a potential conflict of interest.

## Publisher’s note

All claims expressed in this article are solely those of the authors and do not necessarily represent those of their affiliated organizations, or those of the publisher, the editors and the reviewers. Any product that may be evaluated in this article, or claim that may be made by its manufacturer, is not guaranteed or endorsed by the publisher.

## Supplementary material

Supplementary Figure 1
**(A)** Tenfold cross-validation for tuning parameter selection in the LASSO model. The solid vertical lines represent partial likelihood deviance ± standard error (SE) values. **(B)** LASSO coefficient profiles for the 18 DEFRGs.

Supplementary Figure 2 The mRNA expression levels of 10 genes in AML patients (n = 20) and healthy donors (n = 20).

Supplementary Table 1 60 FRGs utilized in this study that were obtained from the previous literature.

Supplementary Table 2 The DEGs between healthy donor and AML patients.

Supplementary Table 3 The DEGs between the low- and high-risk groups.

Supplementary Table 4 The primer sequence of 10 genes in our signature for RT-PCR.
