# Supplementary material for: A novel 10-gene ferroptosis-related prognostic signature in acute myeloid leukemia
Source: Front Oncol. 2022 Oct 20;12:1023040. doi: 10.3389/fonc.2022.1023040 (PMC9630338; doi:10.3389/fonc.2022.1023040)
Supplement: Supplementary Figure 1 — (A) Tenfold cross-validation for tuning parameter selection in the LASSO model. The solid vertical lines represent partial likelihood deviance ± standard error (SE) values. (B) LASSO coefficient profiles for the 18 DEFRGs. [file DataSheet_1.zip › Table S1.DOCX]

**Table S1**

| Ferrotosis-related genes |  | Name |
| --- | --- | --- |
| ACSL4 |  | acyl-CoA synthetase long-chain family member 4 |
| AKR1C1 |  | aldo-keto reductase family 1 member C1 |
| AKR1C2 |  | aldo-keto reductase family 1 member C2 |
| AKR1C3 |  | aldo-keto reductase family 1 member C3 |
| ALOX15 |  | [arachidonate 15-lipoxygenase](https://www.ncbi.nlm.nih.gov/gene/246) |
| ALOX5 |  | [arachidonate 5-lipoxygenase](https://www.ncbi.nlm.nih.gov/gene/240) |
| ALOX12 |  | [arachidonate 12-lipoxygenase](https://www.ncbi.nlm.nih.gov/gene/240) |
| ATP5MC3 |  | ATP synthase membrane subunit c locus 3 |
| CARS |  | cysteinyl tRNA synthetase |
| CBS |  | cystathion ine beta synthase |
| CD44 |  | CD44 molecule |
| CHAC1 |  | ChaC glutathione- specific gamma-glutamyl cyclotransferase 1 |
| CISD1 |  | CDGSH iron sulfur domain 1 |
| CS |  | citrate synthase |
| DPP4 |  | dipeptidyl-dippeptidase-4 |
| FANCD2 |  | Fanconi anemia comple mentation group D2 |
| GCLC |  | glutamate-cysteine ligase catalytic subunit |
| GCLM |  | glutamate-cysteine ligase modifier subunit |
| GLS2 |  | glutaminase 2 |
| GPX4 |  | glutathio ne peroxidase 4 |
| GSS |  | glutathione synthetase |
| HMGCR |  | 3-hydroxy-3- methylglutaryl-CoA reductase |
| HSPB1 |  | heat shock protein beta 1 |
| CRYAB |  | heat shock protein beta 5 |
| LPCAT3 |  | lysophosp hatidylcholine acyltransferase 3 |
| MT1G |  | metallothionein-1G |
| NCOA4 |  | nuclear receptor coactiva tor 4 |
| PTGS2 |  | prostagla ndin-endoperoxide synthase 2 |
| RPL8 |  | ribosomal protein L8 |
| SAT1 |  | spermidine/spermine N1-acetyltra nsferase 1 |
| SLC7A11 |  | solute carrier family 7 member 11 |
| FDFT1 |  | farnesyl-diphosphate farnesyltransferase 1 |
| TFRC |  | transferrin receptor |
| TP53 |  | tumor protein 53 |
| EMC2 |  | ER membrane protein complex subunit 2 |
| AIFM2 |  | apoptosis inducing factor mitochondria associated 2 |
| PHKG2 |  | phospho rylase kinase ,g2 |
| HSBP1 |  | heat-shock 27-k Da protein 1 |
| ACO1 |  | aconitase 1 |
| FTH1 |  | ferritin heavy chain 1 |
| STEAP3 |  | six-transm embrane epithelial antigen of prostate 3 |
| NFS1 |  | cysteine desulfurase |
| ACSL3 |  | acyl-CoA synthetase long-chain family member 3 |
| ACACA |  | Acetyl-CoA carboxylase alpha |
| PEBP1 |  | phosphatidy lethanolamine-binding protein 1 |
| ZEB1 |  | zinc finger E-box-binding homeobox 1 |
| SQLE |  | squalene monooxygenase |
| FADS2 |  | fatty acid desaturase 2/acyl-CoA 6-desaturase |
| NFE2L2 |  | nuclear factor, erythroid 2 like 2 |
| KEAP1 |  | kelch-like ECH- associated protein 1 |
| NQO1 |  | quinone oxidoreductas e-1 |
| NOX1 |  | NADPH oxidase 1 |
| ABCC1 |  | ATP binding cassette subfamily C member 1 |
| SLC1A5 |  | solute carrier family 1 member 5 |
| GOT1 |  | glutamic-oxa loacetic transaminase 1 |
| G6PD |  | glucose-6-phosphate dehydrogenas e |
| PGD |  | phosphoglycerate dehydrogenas e |
| IREB2 |  | iron response element-binding protein 2 |
| HMOX1 |  | heme oxygenase 1 |
| ACSF2 |  | acyl-CoA synthetase family member 2 |
