# Supplementary material for: A novel 10-gene ferroptosis-related prognostic signature in acute myeloid leukemia
Source: Front Oncol. 2022 Oct 20;12:1023040. doi: 10.3389/fonc.2022.1023040 (PMC9630338; doi:10.3389/fonc.2022.1023040)
Supplement: Supplementary Figure 1 — (A) Tenfold cross-validation for tuning parameter selection in the LASSO model. The solid vertical lines represent partial likelihood deviance ± standard error (SE) values. (B) LASSO coefficient profiles for the 18 DEFRGs. [file DataSheet_1.zip › Table S2.DOCX]

**Table S2: The DEGs between healthy donor and AML patients.**

| gene | logFC | pValue | fdr |
| --- | --- | --- | --- |
| ACSL4 | 0.214710881 | 1.38E-15 | 2.09E-15 |
| AKR1C1 | -4.242516965 | 6.46E-33 | 2.18E-32 |
| AKR1C2 | -5.006625304 | 6.46E-33 | 2.18E-32 |
| ALOX5 | 0.421020375 | 7.35E-06 | 8.85E-06 |
| ALOX12 | 0.68413993 | 2.75E-21 | 4.77E-21 |
| ATP5MC3 | -1.036474058 | 6.39E-33 | 2.18E-32 |
| CBS | -7.028377005 | 6.46E-33 | 2.18E-32 |
| CD44 | 0.897777161 | 6.46E-33 | 2.18E-32 |
| CHAC1 | -2.815250832 | 8.26E-33 | 2.44E-32 |
| CISD1 | -1.311304726 | 6.46E-33 | 2.18E-32 |
| CS | -0.066504403 | 8.09E-16 | 1.26E-15 |
| DPP4 | 1.850455788 | 8.05E-15 | 1.19E-14 |
| FANCD2 | -0.591236061 | 2.02E-32 | 5.67E-32 |
| GCLM | -1.216487908 | 4.29E-32 | 1.10E-31 |
| GLS2 | -0.658880382 | 1.00E-10 | 1.32E-10 |
| GPX4 | -0.270459803 | 6.77E-30 | 1.33E-29 |
| HMGCR | -0.722435189 | 6.46E-33 | 2.18E-32 |
| HSPB1 | -0.574307747 | 2.16E-31 | 4.89E-31 |
| CRYAB | -4.92407787 | 6.43E-33 | 2.18E-32 |
| LPCAT3 | -0.116429906 | 4.66E-06 | 5.73E-06 |
| MT1G | -2.59631973 | 2.77E-28 | 5.10E-28 |
| NCOA4 | 0.366738337 | 1.99E-30 | 4.20E-30 |
| PTGS2 | 3.635158351 | 4.65E-29 | 8.86E-29 |
| RPL8 | -0.063642862 | 2.59E-13 | 3.65E-13 |
| SAT1 | 0.224001636 | 3.38E-13 | 4.64E-13 |
| SLC7A11 | -3.230656526 | 6.47E-33 | 2.18E-32 |
| FDFT1 | -0.594786438 | 6.40E-33 | 2.18E-32 |
| TFRC | -0.202414776 | 1.34E-14 | 1.92E-14 |
| TP53 | 1.338688835 | 4.78E-32 | 1.17E-31 |
| EMC2 | -0.626809746 | 7.82E-33 | 2.43E-32 |
| AIFM2 | -1.996895575 | 6.64E-33 | 2.18E-32 |
| PHKG2 | -0.334694732 | 2.22E-25 | 3.98E-25 |
| HSBP1 | -0.771860447 | 6.46E-33 | 2.18E-32 |
| ACO1 | -0.524443785 | 1.81E-31 | 4.28E-31 |
| FTH1 | -0.631412723 | 5.86E-33 | 2.18E-32 |
| STEAP3 | 0.254930027 | 1.31E-06 | 1.64E-06 |
| NFS1 | -0.933609084 | 6.47E-33 | 2.18E-32 |
| ACSL3 | -0.88425775 | 6.46E-33 | 2.18E-32 |
| ACACA | -0.470686058 | 2.87E-32 | 7.68E-32 |
| PEBP1 | 0.144310438 | 5.06E-17 | 8.07E-17 |
| ZEB1 | 0.156611236 | 0.010234146 | 0.011392729 |
| SQLE | -1.161591626 | 6.46E-33 | 2.18E-32 |
| FADS2 | 0.053882255 | 0.019314003 | 0.021102337 |
| NFE2L2 | -0.069031927 | 1.20E-07 | 1.54E-07 |
| KEAP1 | 0.061947388 | 0.000130689 | 0.00015119 |
| NQO1 | -2.153168947 | 6.46E-33 | 2.18E-32 |
| NOX1 | -0.506495041 | 0.004992383 | 0.005664434 |
| ABCC1 | 0.601629279 | 1.71E-30 | 3.73E-30 |
| SLC1A5 | -0.339597098 | 2.59E-30 | 5.27E-30 |
| GOT1 | -0.856435722 | 6.46E-33 | 2.18E-32 |
| G6PD | -0.222153007 | 1.16E-18 | 1.90E-18 |
| IREB2 | 0.103680273 | 7.43E-11 | 9.96E-11 |
| HMOX1 | 2.123163564 | 4.12E-19 | 6.95E-19 |
| ACSF2 | -0.221577826 | 6.54E-05 | 7.71E-05 |
