# Supplementary material for: A novel 10-gene ferroptosis-related prognostic signature in acute myeloid leukemia
Source: Front Oncol. 2022 Oct 20;12:1023040. doi: 10.3389/fonc.2022.1023040 (PMC9630338; doi:10.3389/fonc.2022.1023040)
Supplement: Supplementary Figure 1 — (A) Tenfold cross-validation for tuning parameter selection in the LASSO model. The solid vertical lines represent partial likelihood deviance ± standard error (SE) values. (B) LASSO coefficient profiles for the 18 DEFRGs. [file DataSheet_1.zip › Table S3.DOCX]

**Table S3: The DEGs between the high- and low-risk groups.**

gene logFC pValue fdr

COL9A2 1.224698989 0.004881123 0.015943096

IGF2R 1.307796876 2.04E-06 2.95E-05

EPSTI1 1.009515942 0.002549927 0.009421831

NECAB2 1.735429995 1.59E-05 0.00015303

TMEM105 2.220556752 0.000306058 0.001696682

CLRN1 -1.227914706 0.012605578 0.033837577

CLDN24 -1.395928676 0.019755026 0.048167298

TMEM238 1.101258981 6.23E-08 1.98E-06

ADAMDEC1 2.270978718 0.002157287 0.008234632

TCIRG1 1.243076445 3.01E-08 1.15E-06

CYP4F11 -1.418557967 0.005713664 0.01808098

FXYD6 1.259392889 0.00015324 0.000962389

LMNA 1.330687951 1.92E-05 0.000177124

SULT1C2 -1.318671971 7.07E-05 0.000506783

CTSL 2.075856352 3.82E-07 7.76E-06

KLF4 1.218671609 0.00024556 0.001420164

JAML 1.069793668 5.47E-05 0.000414517

ANO9 1.001531211 0.000128203 0.000832825

CREB3L1 1.107319792 0.003880854 0.013269457

TMEM158 1.170785028 0.020286994 0.049194443

SRGAP1 1.762633996 0.007316104 0.021976808

KCNQ1 1.223423969 6.78E-06 7.78E-05

HHIP -1.63703854 2.60E-07 5.74E-06

MEFV 1.051746091 0.000291582 0.001635862

LRRC9 -1.105025227 7.24E-08 2.19E-06

PATE4 -1.19592021 0.001838492 0.007235777

EPHX2 1.097291979 0.008863157 0.025597243

SSTR2 -1.189158603 8.81E-05 0.000609743

CPA1 -1.032421478 0.000233479 0.001365199

HSPA6 1.24910591 4.47E-05 0.000352746

FEZ1 1.291818065 0.002138158 0.008170669

NDST3 -2.021202574 0.007806278 0.023091967

AC008763.3 1.131244533 0.002164014 0.00825802

NFKB2 1.014239927 7.43E-08 2.23E-06

IFI30 1.39728467 3.96E-05 0.000320234

CCL3 1.117748768 0.00130273 0.005483168

TNFSF12 1.212815321 1.67E-05 0.00015914

PPM1N 1.342298809 5.62E-05 0.000423627

SERPINA1 1.271390108 0.003833165 0.013129376

LDLRAD2 1.000027573 0.000462498 0.002366239

MARCO 1.568301747 0.003050798 0.010936194

OTOF 2.043179218 0.000101826 0.000689654

INA -1.64855662 0.017717871 0.044273789

HBE1 1.364996817 0.000782213 0.003608762

SMIM1 1.130871294 0.001489904 0.006114067

REELD1 1.045188774 0.00963705 0.027390139

TNNT3 1.106940822 0.006448141 0.019968677

SPOCK3 4.229690701 0.001312743 0.005512833

CYTH4 1.036203147 4.06E-07 8.14E-06

AR -1.121040641 0.00463433 0.01533528

STX11 1.005609479 0.000200754 0.00120715

CYTL1 -1.338806822 0.008935106 0.025762278

TCF15 1.691641604 1.09E-05 0.000114179

BMP2 1.788217935 0.007515024 0.022432713

TSPYL5 1.197416908 0.015742861 0.040356095

NRBP2 1.187380732 2.22E-07 5.10E-06

RYR1 1.323405677 0.000105969 0.000710386

PRRT4 -1.713089321 0.000466488 0.002384299

B3GALT1 2.428726854 0.00875535 0.025301803

ADGRB1 1.252077821 0.012815193 0.034291593

TSPAN15 2.098134318 0.001117519 0.004833575

ZSCAN23 -1.141934258 0.001072095 0.004676753

CD300LB 1.425792663 2.29E-06 3.24E-05

ASPG -3.046794211 0.000277975 0.001574583

IGDCC4 -2.846722287 0.000135589 0.000869976

SERPINI1 1.064197101 6.20E-05 0.0004576

BLID -3.112997933 0.000833334 0.003802589

TMEM121 1.342138667 0.008622577 0.025055031

CES1 2.546019999 0.005381862 0.017229964

SECTM1 1.673037847 3.44E-05 0.000286477

G6PD 1.084983054 5.49E-13 1.93E-09

HIST1H3H 1.157432189 0.00509599 0.016478136

GRID1 -1.932885804 0.017409622 0.043669876

LRRC25 1.456466886 7.57E-06 8.51E-05

LGALS3 1.061344686 0.001231446 0.005237864

CHST4 1.005706118 0.019783982 0.048186729

GFRA2 1.070921786 0.000263259 0.001508592

LILRB3 1.001024628 9.82E-05 0.000668341

LAMA3 1.135475842 0.00849729 0.024758863

CCDC42 1.724892762 0.000102725 0.000694345

MX1 1.223984553 0.004539059 0.015072219

CYB5R2 2.173127433 0.00685534 0.020939947

CPE 1.920489707 0.016534832 0.04195687

UGT3A2 -2.767854621 0.010204442 0.028636462

LILRA4 1.231883027 0.01603954 0.040994564

HK3 1.837275638 7.40E-06 8.37E-05

MCOLN1 1.032165484 6.06E-11 1.35E-08

C1QC 3.100818441 0.01525447 0.039323855

TMOD1 1.48558781 0.000191613 0.001160832

CA3 1.010438784 0.012301321 0.033175999

ITGB2 1.2247773 3.51E-07 7.27E-06

ADM 1.098741894 0.01976441 0.048181653

CD4 1.327509234 3.51E-07 7.27E-06

LGALS3BP 1.048528696 0.003228228 0.0114236

PHLDA2 1.075261403 0.002349744 0.00881298

HIST3H2BB 1.014383918 0.016066021 0.041020388

PLEKHN1 1.37389768 0.000337164 0.001830815

GFRA3 -2.671377253 0.002528856 0.009369944

HES5 1.518070617 0.001875454 0.007369154

PPP3R2 -1.223929656 0.005492124 0.017534066

TMEM273 1.031031441 9.82E-05 0.000668341

PLXNB3 1.234801343 0.000663994 0.003178139

CDC42EP3 1.066649073 0.000283865 0.00160136

PDGFB 1.1861066 0.012875482 0.034421238

SFTPD 1.394003799 0.001114054 0.004827685

SHISA4 1.726635594 5.05E-07 9.73E-06

GABRE -3.139651062 0.018954793 0.04668715

PHACTR3 2.514671647 0.005691772 0.018021111

LST1 1.006894343 1.28E-05 0.000129121

IFITM3 1.109654114 0.012901571 0.034457878

SLC35G3 -1.315824669 0.000363236 0.001940266

SNAP25 2.685172961 0.006442595 0.019962482

AL662899.1 1.606336152 0.009229454 0.026499574

MAFB 2.208083876 1.28E-05 0.000129121

FRMD7 -1.18991011 0.00030151 0.001677543

SPAG17 1.504204034 0.019466318 0.047631971

ST14 1.629175757 1.62E-06 2.47E-05

SEMA3C 1.435942161 0.002471861 0.009195947

ASGR2 1.344489218 0.003398519 0.011928151

NWD2 -1.266566097 0.006157872 0.019266601

SHANK1 -1.08527068 0.000742106 0.003459934

SH3TC1 1.228111111 7.83E-08 2.30E-06

BEX2 1.424127269 0.002077079 0.007993483

PEAR1 1.142936163 0.015563758 0.039936667

BCL2A1 1.267897329 1.88E-06 2.78E-05

TUBB2B 1.171318237 0.01431183 0.037360773

SMPDL3A 2.053634591 0.000935535 0.004192562

GGT6 -1.148183222 0.000806825 0.003707209

GATA1 1.410166704 0.000711204 0.003350436

CCDC151 1.01774555 0.01440839 0.037578566

ALOX5AP 1.238550729 9.00E-05 0.000620177

SLC11A1 1.276189449 0.000206379 0.001231839

ITLN1 1.132131891 0.00066497 0.003178568

RTN2 1.29907986 1.42E-06 2.22E-05

NEURL1 1.173075786 3.70E-05 0.000301029

IDO1 1.418604114 0.00103142 0.004526551

C1QB 3.439267454 0.014215548 0.03714658

CMTM5 2.440236759 0.000364524 0.001944807

THBS1 3.207066302 9.20E-06 9.93E-05

AMHR2 1.060730348 0.000422342 0.002201358

HOXB5 1.631480705 0.000143085 0.00090794

TEX101 -1.467553513 3.75E-05 0.000304849

KLK1 1.263779627 0.001602461 0.006492794

AFF2 -1.858348997 0.001098132 0.004768059

FOXD1 1.70561624 0.012774613 0.034204678

PATE3 -1.450165723 0.004976452 0.016191686

PCOLCE2 1.433018318 0.004942616 0.016108227

FGF13 -2.444123913 0.008469417 0.024687832

UTF1 2.575367344 7.48E-08 2.24E-06

GSTT2B 1.311201688 0.003061606 0.010958004

TIMP4 1.19773595 0.005507435 0.017574803

TMPRSS11D -1.069992165 7.07E-06 8.05E-05

HSPB7 1.898389998 0.012860003 0.03438653

KLHDC8A 1.982151999 0.015973474 0.040840864

LILRB4 1.224498944 2.38E-05 0.000211637

HOXA4 1.008895373 0.000600122 0.002935824

PSAT1 1.023056619 3.69E-05 0.000300879

FBP1 1.568192572 0.000513344 0.002587934

KCNC3 1.695471783 0.000664209 0.003178139

LAG3 1.2998862 0.01191275 0.032365629

SYT1 1.766120476 0.020320274 0.049266471

IL31RA 1.036884156 0.007667286 0.022808146

HES4 2.717978097 0.00060252 0.002946508

HBQ1 1.058359259 0.016637711 0.042132556

RD3L -1.770654818 0.010284377 0.028813826

GZMB 1.21325418 0.000327449 0.001789376

GNG4 -2.122619287 0.013966501 0.036613228

SLC35E4 1.003279511 1.06E-05 0.000111283

RHAG 1.484123546 0.013590953 0.035838694

HOXA7 1.196058642 2.55E-05 0.000223908

CRYBA4 1.642447995 0.012893232 0.034457878

SLC2A12 -1.444158265 0.007782017 0.023045721

C5AR1 1.737198631 2.56E-05 0.000223908

PNPLA6 1.029880688 2.01E-11 7.30E-09

ARHGAP6 1.546497764 0.009357696 0.026784046

MYOF 1.383321654 4.22E-05 0.000337526

ULBP1 1.518031493 0.000121287 0.00079564

LAMP5 1.100264421 0.009416803 0.026914082

HGF -1.716618038 0.000113343 0.000750681

CAMK1 1.203940131 0.000953858 0.004248819

KCNA7 -1.617567166 0.011171319 0.030687156

HOXB2 1.070878893 0.000564311 0.002798982

MPO -1.187614782 0.015866714 0.04059804

C6orf223 2.438518933 0.00022026 0.001296159

GPC3 3.087082233 8.05E-06 8.96E-05

SORT1 1.404924676 8.44E-06 9.25E-05

COPZ2 1.093832741 0.000738412 0.003451767

MRC1 1.570710919 0.01916982 0.04709339

C1orf162 1.001739831 0.001672217 0.006725973

HOXA3 1.004490669 0.000354487 0.001904161

ADGRE1 1.174181232 6.28E-05 0.000461756

EPB41L3 2.939448 8.40E-05 0.000586396

PARP3 1.034983834 1.06E-09 9.26E-08

CD40 1.03158489 0.000228135 0.001338494

AL645941.2 1.071660985 0.000773716 0.003575439

CCDC170 1.150112893 0.000457304 0.002348053

IL1RL2 1.084888898 0.008935262 0.025762278

TBC1D12 1.505529825 0.001260176 0.005339953

ITGAX 1.299033636 1.41E-07 3.62E-06

PI4K2A 1.064460525 2.61E-05 0.000227275

LILRA1 1.166896216 9.40E-06 0.000101104

KHDRBS2 1.170457459 0.002920349 0.010548173

TP53INP2 1.903290498 0.000870644 0.003949419

FCGR3A 2.512603476 0.00167207 0.006725973

VNN1 1.045724389 0.000212064 0.001258616

CLIC6 2.130452822 0.000210144 0.001251369

SIX4 -2.675801731 0.000585059 0.002878492

GPRIN1 1.068136876 8.56E-06 9.36E-05

SH3BP5 1.036080045 6.03E-05 0.000446963

HIST3H2A 1.107160423 0.000371505 0.001977532

PNMA2 -1.526448875 0.000308116 0.001704663

HIST2H3D 1.146015024 0.01986146 0.048366885

HLA-DQB2 1.369715377 0.001018647 0.004480406

CAVIN2 1.053026578 0.001973553 0.007666611

ZNF703 1.138617598 0.012978315 0.03462358

MORC1 1.577121748 0.004889968 0.015966862

MMP7 4.917679543 5.75E-06 6.85E-05

GADL1 -1.392317423 0.001887913 0.007405436

LYNX1 2.10598653 6.96E-06 7.95E-05

UPK3A 1.072843827 0.000342729 0.001853722

RUFY4 1.6023167 0.000235595 0.001375239

VNN2 1.143231159 0.000116607 0.000770445

ITGB6 -1.305039081 0.002458531 0.009161139

FGD2 1.147616327 7.82E-06 8.74E-05

ITGA7 1.274792249 0.000220038 0.001295507

CD163 3.08266495 0.002342804 0.008790192

SAMD4A 1.042073252 0.005205958 0.016760012

RRAD 1.893428336 1.16E-05 0.000120011

ADAMTS18 -2.163548211 0.000267113 0.001527501

TNNI2 1.856241933 1.10E-07 3.01E-06

CCL22 1.551460757 0.000201021 0.001207706

OR14A2 -1.04580369 0.017320316 0.043508478

TRPM6 -1.545774602 0.00282475 0.010253955

TSPO 1.02136314 1.01E-06 1.71E-05

KCNE1 1.787156027 4.30E-05 0.00034246

FHL2 1.495056584 0.00084101 0.003832249

MS4A14 1.115608056 0.007367746 0.022091221

PTAFR 1.280977342 2.04E-05 0.000185945

SPOCK1 2.142252583 0.003116677 0.011134686

ZNF214 1.181897643 0.006776367 0.020749329

NCF1 1.101105225 5.05E-05 0.000388384

FAM198A -1.586711222 0.010103701 0.028434849

EBI3 2.517176142 4.32E-05 0.000343483

AIFM2 1.07617168 7.92E-08 2.30E-06

BATF3 1.147787485 1.69E-05 0.00015995

CEBPB 1.334552311 2.02E-06 2.93E-05

CXCL16 1.312742135 0.000504481 0.002548547

KIAA0408 -1.064130337 0.00150627 0.006168845

TMEM200A 2.40260805 0.004217275 0.014236015

PWP2 1.061196683 0.002874565 0.010410095

TCN2 1.265562985 0.004916129 0.016029141

ACSM1 -1.10602284 0.000986146 0.004366594

ITGB7 1.011477814 9.74E-08 2.73E-06

CD70 1.141702011 0.017839658 0.04448129

SIGLEC1 1.700331162 0.003718867 0.012818757

PID1 1.781248946 0.002830319 0.010271462

RASEF 2.399659398 0.020141431 0.048926417

C10orf105 1.369321121 5.58E-07 1.04E-05

ZFPM1 1.045260546 6.99E-05 0.000502612

AIM2 1.270182724 0.000604472 0.002951173

S100A6 1.377118869 3.21E-08 1.19E-06

CD207 2.004187797 0.019218756 0.047193864

FGR 1.365947552 8.17E-06 9.04E-05

TNFRSF1B 1.092009714 3.15E-06 4.13E-05

AC018523.2 -1.007417472 0.003702848 0.012778166

PF4 1.185765403 0.000341976 0.001853722

SLC38A4 -1.293781674 0.000245295 0.001420164

TNFSF9 1.231685102 0.001152231 0.004960197

CLCN1 1.190707485 0.000105284 0.000706816

CD300C 1.368317561 3.37E-06 4.39E-05

C11orf97 -1.131397889 0.000128168 0.000832825

CHAT -2.034269902 0.014110302 0.036940995

BLVRB 1.250613203 7.52E-07 1.35E-05

CAVIN3 2.234940193 0.006726892 0.020649927

MT1E 1.202963126 0.0012667 0.005361001

SLC9A9 1.012650305 0.001107051 0.004801261

RAB11FIP5 1.530783467 0.000535664 0.002685287

ODF3L1 1.148416326 0.000978334 0.004335296

SCPEP1 1.181558566 0.008694649 0.025173916

EPOR 1.571302008 5.94E-06 7.04E-05

COMTD1 1.22517323 2.50E-12 2.46E-09

VENTX 2.299863858 2.65E-06 3.66E-05

NUDT14 1.03027931 7.79E-07 1.38E-05

EFNB3 -2.44889111 0.009083409 0.026135644

ZMYND15 1.185395211 0.005040684 0.016338933

IL12RB1 1.062392362 2.45E-09 1.75E-07

HOXA5 1.023621383 0.00035714 0.001914383

CDC42EP1 1.274974909 0.001311753 0.005512833

HELZ2 1.019604811 6.28E-06 7.32E-05

DDIT4 1.000039014 1.34E-06 2.12E-05

RSPO2 1.681856853 0.015176908 0.039160346

HTR3B -1.210896782 1.63E-05 0.000155682

TUBAL3 2.401426015 0.020613214 0.049818883

MSR1 2.280745665 0.000268228 0.001532884

MSMB -1.063502942 0.019375853 0.047486524

ANKRD65 1.499069435 0.003863981 0.013215044

CES4A 1.501250122 6.01E-06 7.10E-05

ADAMTS15 -2.038043704 0.003887153 0.013279196

SLC15A5 -1.561878888 0.000431032 0.002236063

LILRA6 1.218884764 6.03E-05 0.000446963

CCNJL -1.532865503 0.010048164 0.028330641

PDE4A 1.344278057 7.65E-06 8.58E-05

RASL12 -4.581571912 0.017185251 0.043233021

CELSR2 1.027142375 4.47E-08 1.53E-06

VASH2 -1.102505965 0.001806872 0.007141249

SCO2 1.122984621 1.71E-09 1.32E-07

SPINT1 1.157755659 7.71E-07 1.37E-05

ADAP2 1.070029118 5.25E-05 0.00040205

SNCG 1.343698718 0.00120702 0.005152746

SLC28A3 -1.13986119 0.016058481 0.041020388

GOLGA6L7 1.20314772 0.009165383 0.026332077

MYBPHL 1.774046246 0.000189474 0.00115037

ZNF385A 1.135660036 1.09E-06 1.79E-05

SIGLEC11 1.568197973 0.000570174 0.002821379

SDC4 1.08036544 0.00133536 0.005587053

UPP1 1.266379211 0.000705397 0.003329126

IFITM10 1.075789223 0.004154526 0.014061651

SERINC2 2.318107409 3.07E-06 4.08E-05

DPP4 1.157188052 0.001618856 0.00654346

C4orf48 1.037675066 4.02E-06 5.07E-05

ESPN 2.291846756 0.00163856 0.006615711

PKIB 1.029121079 6.25E-05 0.000460769

FTH1 1.159539552 2.06E-09 1.56E-07

ATP6V0E2 1.094393053 7.15E-10 6.98E-08

SOCS2 1.290996949 0.00386142 0.013210562

BFSP1 1.39446786 7.90E-06 8.81E-05

SPATS2L 1.271344563 2.31E-06 3.27E-05

CALCA -1.08813091 0.001438264 0.00594287

PYCARD 1.141727403 1.53E-10 2.57E-08

FN3K 1.004887907 0.002269839 0.008582883

NECTIN4 1.907027793 4.13E-05 0.000331764

GDF15 1.704126981 1.51E-05 0.000147594

UNC93B1 1.090908557 1.32E-07 3.45E-06

LOXL4 -1.556497951 0.000423594 0.002205427

SCRN1 1.00213059 0.006823217 0.020853393

EFCC1 1.357868725 0.011917857 0.032365629

HOXB1 2.181329765 0.001251588 0.005305829

PTP4A3 1.248793903 3.29E-06 4.31E-05

OXGR1 1.566583971 0.000219136 0.001291748

SLC43A2 1.064446837 1.27E-05 0.000128305

SNPH 1.187874782 3.58E-05 0.000294444

SEL1L2 2.278386668 0.000361305 0.001931535

C16orf45 1.039741885 8.23E-06 9.10E-05

SRPX2 1.295590131 0.002138762 0.008170712

CLCNKA 1.660051814 4.24E-05 0.000339571

ITGB3 1.25683181 0.006916637 0.021072315

LTA 1.028049143 0.002948123 0.010635786

PPDPF 1.075029024 5.03E-11 1.17E-08

NPL 1.096916883 0.000841977 0.003832249

SLC1A2 -1.1138199 0.004542619 0.015079307

GATA5 2.462478494 0.011571995 0.031598772

TNK1 1.947885464 0.015158528 0.039152463

SYT17 1.793809695 0.000350712 0.001889484

TRPM4 1.229849511 0.000139237 0.000888433

LY6G6F 1.360608563 0.008348428 0.024386757

KIR2DL1 1.109624491 0.012233826 0.033034218

BATF2 1.612609898 3.83E-05 0.000310608

GDPD4 1.466391087 0.000475739 0.002423737

GADD45G 1.480027661 5.12E-05 0.000392916

FOLR2 1.615253858 0.000472711 0.002413663

GPR35 1.115026755 1.26E-05 0.000127423

RAB17 1.288386077 0.001458905 0.006011361

ALDH1A1 1.290885822 0.002794193 0.010158483

FCN1 1.076985727 0.000653388 0.003138502

C3 1.378754241 0.000449865 0.002315031

CDKN1A 1.183521429 0.000814282 0.003729359

PRR7 1.069961038 7.34E-05 0.000522039

IL10 2.68397951 0.012240399 0.033034218

HSD3B7 1.041673999 0.000202331 0.001213578

OASL 1.186349197 0.00215008 0.008209398

KCNE1B 2.192508865 0.001901299 0.007443108

ABHD11 1.004115681 2.88E-10 3.79E-08

HCK 1.10796215 1.80E-06 2.68E-05

ZNF503 1.437766222 0.000773967 0.003575439

TGM2 1.647167145 4.55E-06 5.59E-05

ISG15 1.128342895 2.48E-06 3.46E-05

MPP7 1.01555541 1.78E-05 0.000166959

SLC24A3 -1.23046686 0.000841645 0.003832249

IL4I1 1.413322696 9.89E-08 2.76E-06

CCL23 2.470407214 6.98E-06 7.97E-05

CTSV 3.07218261 0.001238167 0.00526288

AC092042.3 -1.45490196 0.003333413 0.011720544

CRYGD 1.215467472 0.001758956 0.006999243

SAGE1 1.300408392 0.002376389 0.00889596

SLC35G1 1.257874955 3.10E-06 4.10E-05

AL157935.2 1.029698353 1.39E-05 0.000137345

CCR5 1.007421935 6.46E-05 0.000472327

ETV7 1.353392864 0.006378684 0.019786703

COL2A1 -3.208367603 0.000187586 0.001142432

NR4A1 1.310438924 0.006776069 0.020749329

MMEL1 1.166625354 0.003752227 0.012919441

PATE2 -1.368151112 0.0163323 0.041550094

ALDH3A1 1.257588454 0.007514037 0.022432713

C1QA 3.805756491 0.000564301 0.002798982

PNPLA3 1.287304365 0.00032769 0.001789376

ITGAM 1.310511613 3.37E-06 4.39E-05

TNFAIP2 1.053068469 1.69E-05 0.00015995

KIR2DL3 1.139756562 0.005302677 0.017012036

MEGF10 -1.516826928 0.017000525 0.042870077

ASB2 1.268665011 6.18E-09 3.50E-07

H1FX 1.112750607 0.001324913 0.005549407

TMTC1 2.655024375 0.002656194 0.009744802

TMEM63C 2.018389774 3.71E-06 4.76E-05

MOCOS 2.132595751 2.88E-07 6.21E-06

VSIR 1.560373981 2.31E-09 1.69E-07

CRISPLD2 1.136381687 0.013241666 0.035132177

C1orf127 1.220978707 1.50E-05 0.00014654

ELANE -2.134011522 0.008402 0.024512322

CDH23 1.035394545 1.10E-06 1.81E-05

SHTN1 1.160396969 0.000442523 0.002287887

CTSD 1.364093073 1.07E-07 2.92E-06

KIR2DL4 1.822794136 4.38E-05 0.000347061

PSCA 1.216088385 0.006571883 0.020285516

CDCP1 1.236433988 0.000559573 0.002779905

TRPV4 2.643269211 0.00173282 0.006911212

F2RL3 1.412098529 0.020345982 0.049320121

SLC15A3 1.18048274 2.51E-05 0.000220441

IL2RA 1.376568053 0.000315996 0.001738842

C22orf42 1.010569242 0.001502775 0.006161379

HMOX1 1.841537508 1.25E-06 2.00E-05

VWF 1.729886418 0.005208969 0.016760012

ENPP2 1.072589079 0.012900093 0.034457878

C3orf56 -1.63840176 0.000268277 0.001532884

PTGFR 1.772430275 0.002468391 0.009192903

TYMP 1.386341239 3.70E-05 0.000301029

APOBEC3A 1.469249489 0.000367785 0.001960694

LILRB1 1.394375076 1.46E-05 0.000143307

IL1RN 1.168223346 0.000224074 0.001316613

FPR1 1.474505568 0.000658907 0.003158565

PDZK1IP1 1.219191629 0.017715956 0.044273789

KRT81 1.489038081 0.015527357 0.039870433

CYP3A43 -1.038381092 5.83E-06 6.93E-05

MYT1L -4.623183848 0.006575719 0.020292809

CASP5 1.231556174 0.004503809 0.014983949

KCTD17 1.058870978 3.77E-07 7.71E-06

MAP1LC3A 1.032616183 0.010927601 0.03018016

HTR7 1.78934147 9.24E-06 9.96E-05

PLD3 1.078814866 5.04E-06 6.09E-05

ADAM8 1.106389866 1.43E-05 0.000140803

LDLRAD3 1.99709811 5.47E-05 0.000414517

FBLN2 1.376220755 0.007157837 0.021638437

B3GAT1 1.013888868 0.006858687 0.020945526

HOXB7 1.420116139 0.015090595 0.039003384

HOXA9 1.027930586 0.000539853 0.002699683

CD200R1 1.003950788 0.000748194 0.003478888

LY86 1.141878683 0.000140909 0.000895463

DKK2 1.354213027 0.003182823 0.011294841

FCER1G 1.054654023 0.001293034 0.00544427

GCKR 2.350734943 0.001152433 0.004960197

ST6GALNAC1 1.063740683 0.008436007 0.024606063

ABCC3 1.515143233 0.001593154 0.006460708

CHCHD10 1.216546251 4.26E-10 5.06E-08

SGMS2 1.248564331 0.000387898 0.002047205

UBXN10 1.451186503 0.000710019 0.003349733

PPFIBP2 1.144577686 2.41E-05 0.000213439

HNMT 1.685229875 0.000848894 0.003858883

ALPP 2.267878378 1.49E-05 0.000145957

IFI27L2 1.04895689 6.36E-08 2.00E-06

ANKRD55 1.077619321 2.21E-05 0.000199733

KAZALD1 1.837892029 1.28E-05 0.000129121

DNAJC5B 1.777781807 0.007486931 0.02236339

TIMP3 2.494764692 0.004494145 0.014968893

CRIP3 1.208698489 7.16E-05 0.000511631

VDR 1.29049518 3.68E-06 4.74E-05

GNGT2 1.331640914 6.83E-08 2.11E-06

GHSR -1.459431619 0.004142651 0.0140357

NKX2-3 1.380911169 0.006286073 0.019574354

TFEB 1.215727243 1.11E-08 5.49E-07

PURG -2.60694087 0.002504452 0.009294538

CXCL10 1.447238196 0.000151126 0.000951513

BAG3 1.712415046 5.55E-06 6.66E-05

ANKRD9 1.451587338 6.86E-08 2.12E-06

SERPINE1 -1.172404525 0.012000633 0.032541155

LSP1 1.165407748 5.57E-06 6.66E-05

CRIP1 1.370879216 1.88E-07 4.58E-06

CD300E 1.08663763 0.000123323 0.000806877

GABRR3 -1.185214872 0.011396739 0.031206814

ZNF532 1.483519578 0.000123408 0.000806877

MISP -1.03970971 0.00017099 0.001055357

PLG -1.030373649 0.000716377 0.003372801

RNASE1 1.560819819 0.020520282 0.04963254

HOXB6 1.401534787 0.000330351 0.001801821

GPR25 2.819345123 0.00265849 0.009748031

TMIGD3 1.225041607 0.003915559 0.013361065

HIST1H1C 1.831227712 0.002773751 0.010089727

MCOLN2 1.460287752 0.016365942 0.04159506

CD1D 1.363009511 3.38E-05 0.000281523

ATP12A -1.273735148 0.012999529 0.034651994

ABI3 1.501473653 3.59E-07 7.40E-06

CXCL11 1.721424743 0.004305433 0.014473885

SIRPD 1.040647166 0.003770653 0.01297964

METTL7B 1.925148496 2.53E-06 3.52E-05

LGALS1 1.296315234 9.00E-07 1.56E-05

PHOSPHO1 1.202194293 0.000526822 0.002648038

CLCNKB 1.250961574 0.007362335 0.022091221

EGR2 1.154744081 0.018934376 0.046650417

NTNG2 -1.42342291 0.005097983 0.016478136

MYO7A 2.137008548 5.11E-07 9.78E-06

IL1R2 3.353503001 0.00186112 0.007317005

KRTAP10-3 1.321928095 3.47E-06 4.49E-05

OPLAH 1.164309699 6.27E-06 7.32E-05

SIX3 -3.765084564 0.000134224 0.000864239

TMPRSS11A -1.199637665 0.000201543 0.001210313

MVP 1.106541415 1.48E-09 1.20E-07

CCR1 1.078566715 0.000384561 0.002037656

CD14 1.234725298 0.000176348 0.001082738

MARCKS 1.130622405 6.09E-05 0.000450244

TMEM74 1.236298023 0.006036578 0.018951663

RASL11A 1.036022421 0.003223446 0.011409609

SLC2A14 1.862290406 0.003715576 0.012818757

ECHDC3 1.311058786 1.28E-06 2.04E-05

GRK1 -1.744890835 0.009054551 0.026072016

KCNJ6 -1.204483411 0.017541065 0.043935584

STOX2 -1.542707429 0.007512604 0.022432713

DPPA4 1.056087821 0.016747423 0.042332568

F12 1.579199692 3.13E-07 6.62E-06

OXCT2 1.006445409 0.003019818 0.010842066

RAB31 1.132129941 3.14E-05 0.000264949

CX3CR1 1.451092433 3.74E-05 0.000303715

C1QL1 1.930349568 0.008106203 0.023820282

TDRP -1.152127458 0.001482065 0.006088718

C19orf84 1.025713184 0.012285632 0.033140174

CTAGE8 1.181606806 0.003388903 0.011897432

TMEM212 -1.407375798 0.00176884 0.007026394

EXOC3L4 1.382629264 0.000247702 0.00143137

SYTL4 1.153395509 6.39E-05 0.00046905

SAT1 1.187856102 0.000296939 0.001660161

DUSP8 1.808493143 0.001236945 0.005259308

LILRB2 1.646174089 1.07E-06 1.78E-05

GLB1L2 1.044573689 0.000377215 0.002005599

CDHR1 1.126549914 0.001111752 0.004819223

ULBP3 1.685547613 1.28E-07 3.36E-06

LY6G6E 1.292304689 0.002339341 0.008785922

HRH1 1.516708285 0.000473833 0.002418498

RRAS 1.282865924 1.26E-05 0.000127423

ISL2 1.600392541 0.004273447 0.014402447

CBR1 1.164472445 2.72E-11 8.10E-09

STAC 1.220493388 0.00683732 0.020889536

CST3 1.024991521 0.001028193 0.004513826

NINJ1 1.300614681 2.91E-07 6.27E-06

CD36 1.121464389 0.000120008 0.000789886

JPH4 1.133674637 1.45E-05 0.000143307

PPM1M 1.027767195 1.24E-08 5.81E-07

MYRFL -3.02522181 0.000537454 0.002690393

SIGLEC7 1.350489645 7.93E-05 0.000558643

PLEKHH3 1.036256162 0.000119928 0.000789886

KIAA0895 -1.045900851 0.001725222 0.006886817

DHRS9 1.373230625 0.000130648 0.000845571

PDK4 1.917133902 0.005881315 0.018529919

PLCH1 -1.732599718 0.005554448 0.017700073

PRICKLE2 2.870648348 0.009093788 0.02615912

LPO -1.335537914 0.003203894 0.011347119

PPBP 1.507728071 0.002731446 0.00996572

VMO1 2.156628179 0.000200905 0.001207533

LILRA5 1.512061349 6.21E-05 0.000458001

GPBAR1 1.473099948 0.000182932 0.001118539

DTX3 1.116588432 0.010086429 0.028411646

ITGA2B 1.484503621 0.020402975 0.049381737

DOK2 1.260035825 3.34E-07 6.96E-06

EPS8L3 1.025840005 0.011500177 0.03145254

SCIMP 1.055445439 0.000252302 0.001453804

HOXA10 1.009844249 0.000508268 0.002565741

CPA3 -1.187305209 0.00975909 0.027657112

PGC 1.774249769 0.000104122 0.000700723

APOBEC3H 1.191723304 2.66E-05 0.00023083

GPR20 1.736965594 0.000696953 0.00330393

CDA 1.128747219 0.000710881 0.003350436

PRDM16 1.197835855 0.000555286 0.002761596

CD101 1.318333512 0.000345664 0.001866317
