# Supplementary material for: A novel 10-gene ferroptosis-related prognostic signature in acute myeloid leukemia
Source: Front Oncol. 2022 Oct 20;12:1023040. doi: 10.3389/fonc.2022.1023040 (PMC9630338; doi:10.3389/fonc.2022.1023040)
Supplement: Supplementary Figure 1 — (A) Tenfold cross-validation for tuning parameter selection in the LASSO model. The solid vertical lines represent partial likelihood deviance ± standard error (SE) values. (B) LASSO coefficient profiles for the 18 DEFRGs. [file DataSheet_1.zip › Table S4.DOCX]

**Table S4. The primer sequence for RT-PCR.**

| Gene | Forward sequence | | Reverse sequence |
| --- | --- | --- | --- |
| **CD44** | | 5'- ACACGAAGGAAAGCAGGACC -3' | 5'- CCCATGTGAGTGTCCATCTGA -3' |
|  | |  |  |
| **CHAC1** | | 5'- GCAGGGAGACACCTTCCATC -3' | 5'- AGGTAACCAGGGTTCTGCTCC -3' |
|  | |  |  |
| **CISD1** | | 5'- TTGGAGGTCCAAAAAGTTCCCA -3' | 5'- ATCAGAGGGCCCACATTGTC -3' |
|  | |  |  |
| **PDD4** | | 5'- ATGCCAGGAGGAAGGAATCT -3' | 5'- TCCGGATTCAGCTCACAACTG -3' |
|  | |  |  |
| **NCOA4** | | 5'- TCGAACTCCTCTACAGGCATA -3' | 5'- TGGGATCTGAAAATTCCCAACG -3' |
|  | |  |  |
| **STA1** | | 5'- AAGATGGTTTTGGAGAGCACC -3' | 5'- CCAACAATGCTGTGTCCTTCC -3' |
|  | | ' |  |
| **SLC7A11** | | 5'- TGACTGGAGTCCCTGCGTAT -3' | 5'- TGTTCTGGTTATTTTCTCCGACA -3' |
|  | |  |  |
| **AIFM2** | | 5'- GCAAAGCGTTTGAGAGCAGA -3' | 5'- CAATGGCGTAGACGTTGCTG -3' |
|  | |  |  |
| **G6PD** | | 5'- GACGACGAAGCGCAGACAG -3' | 5'- CCGACTGATGGAAGGCATCG -3' |
|  | |  |  |
| **ACSF2** | | 5'- TCTGCAGCAGCACATTCCAGA -3 | 5'- GGTGCACCCCTGAACGTAG -3' |
|  | |  |  |
| **GAPDH** | | 5'- AATTCCATGGCACCGTCAAG -3' | 5'- GAGGGATCTCGCTCCTGGAA -3' |
