# Supplementary figures and images for: A novel 10-gene ferroptosis-related prognostic signature in acute myeloid leukemia
Source: Front Oncol. 2022 Oct 20;12:1023040. doi: 10.3389/fonc.2022.1023040 (PMC9630338; doi:10.3389/fonc.2022.1023040)

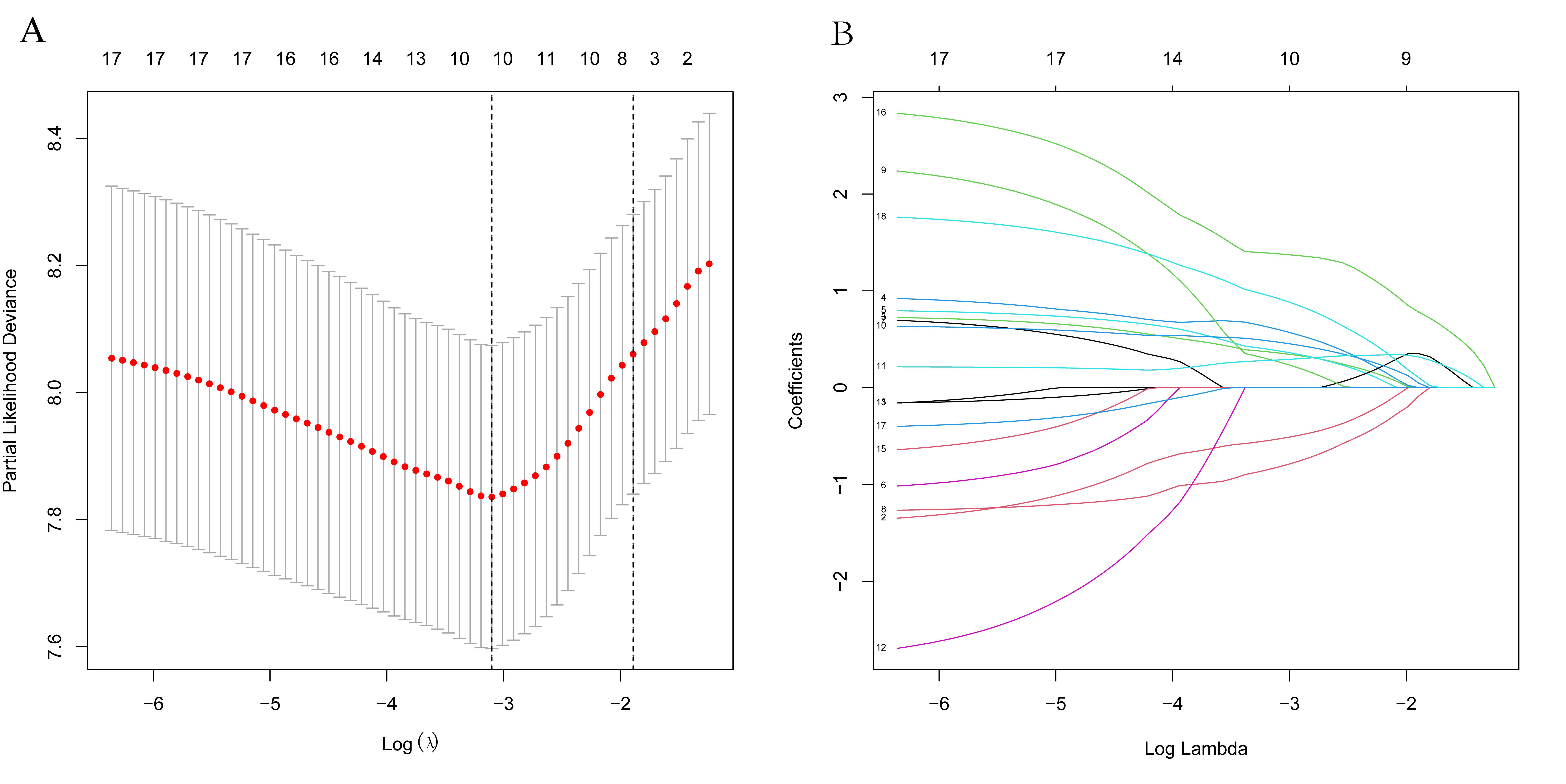

Supplement: Supplementary Figure 1 — (A) Tenfold cross-validation for tuning parameter selection in the LASSO model. The solid vertical lines represent partial likelihood deviance ± standard error (SE) values. (B) LASSO coefficient profiles for the 18 DEFRGs. [file DataSheet_1.zip › Figure S1.TIF]

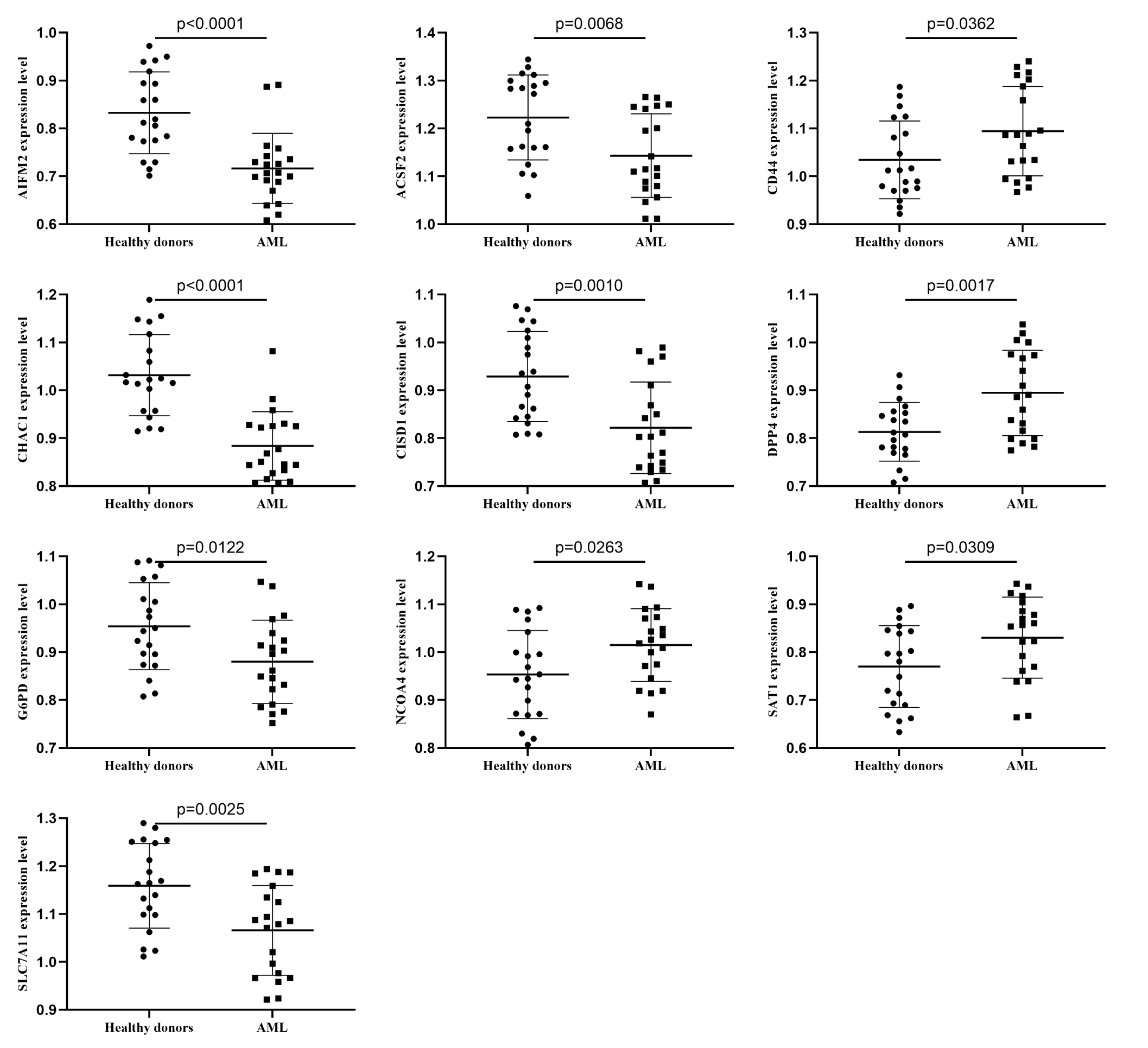

Supplement: Supplementary Figure 1 — (A) Tenfold cross-validation for tuning parameter selection in the LASSO model. The solid vertical lines represent partial likelihood deviance ± standard error (SE) values. (B) LASSO coefficient profiles for the 18 DEFRGs. [file DataSheet_1.zip › Figure S2.TIF]
